# MGA: a tool for haplotype-mixed assembly of long and accurate reads

**DOI:** 10.1186/s13059-026-04128-5

**Published:** 2026-06-17

**Authors:** Zhenmiao Zhang, Marcus W. Fedarko, Anton Bankevich, Pavel A. Pevzner

**Affiliations:** 1 Department of Computer Science and Engineering, University of California San Diego, La Jolla, USA; 2 Department of Computer Science and Engineering, Pennsylvania State University, University Park, USA

**Keywords:** Genome assembly, Long reads, De Bruijn graphs

## Abstract

Recent large-scale genome sequencing projects have generated near-complete diploid assemblies that reconstruct both haplomes. Producing such assemblies remains formidable, typically requiring large teams, extensive manual curation, and integration of multiple sequencing technologies. However, for many species and applications, a near-complete haplotype-mixed assembly—representing a mosaic of both haplomes—provides most of the same benefits for downstream analyses. Such assemblies can be generated automatically at lower cost using only HiFi reads. Here we present Mosaic Genome Assembler (MGA), a tool that generates near-complete haplotype-mixed assemblies from HiFi reads alone. We show that MGA substantially outperforms existing haplotype-mixed assemblers.

**Supplementary Information:**

The online version contains supplementary material available at 10.1186/s13059-026-04128-5.

## Background

### From haplotype-mixed assemblies to diploid assemblies

Until recently, most sequencing efforts were targeted at the generation of haplotype-mixed *consensus assemblies*, representing a mosaic of two haplomes [1–3]; diploid assemblies accounted for only a small fraction of sequencing projects. This situation has changed with the advent of HiFi reads, which enabled the generation of telomere-to-telomere (T2T) diploid assemblies. In 2022, the T2T consortium assembled the first complete human genome, ushering in the era of “complete genomics,” which aims to produce complete assemblies of diverse genomes [4]. In 2023, the Human Pangenome Reference Consortium generated T2T diploid assemblies of 47 human genomes [5] with the goal of assembling 350 such genomes by 2026.

We distinguish between a *T2T diploid assembly*, which represents both haplomes, and a *T2T consensus assembly*, which represents a haplotype-mixed patchwork of the two “true” haplomes. Although T2T diploid assemblies are valuable for medical research, they are often less important for studies of non-human genomes. Moreover, constructing such assemblies remains a formidable task that typically requires large teams, multiple sequencing technologies, and time-consuming manual analysis [4, 6, 7]. Despite these additional costs, T2T diploid assemblies can still contain structural errors—a recent reanalysis of T2T assemblies generated by the Vertebrate Genome Consortium revealed that many of them misassembled a short immunoglobulin heavy chain locus [8]. These considerations raise the question of whether the advantages of producing a T2T diploid assembly of a non-human genome truly outweigh the additional costs compared with a T2T consensus assembly. We note that some large-scale sequencing projects prioritize the generation of a single reference genome rather than fully resolving both haplotypes. For example, the primary goal of the Earth Biogenome Project (EBP) is “*to obtain for each species a sufficiently complete, accurate, and contiguous representative genome sequence that can provide a reference for further genomic analysis,*” while the generation of alternate haplotypes is considered a secondary goal [9, 10].

Another consideration is that no truly complete T2T diploid assembly has been generated yet—all T2T assemblies reported to date [4, 5, 7, 11–13] failed to assemble certain highly repetitive regions (HRRs), such as rDNA repeats. Even in smaller fungi genomes some HRRs remain unassembled despite using state-of-the-art assembly tools that integrate multiple sequencing technologies [14, 15]. Because HRRs are usually analyzed only in specialized repeat-focused studies [16] and because aligning reads to these regions involves various challenges [17, 18], it is often unnecessary to devote substantial effort to fully assembling them. Moreover, HRRs show extensive variability across the population, with some centromeres differing by several megabases in length between human haplotypes [19]. These variations suggest that knowledge of a single individual’s two haplomes may not be very useful for representing the true diversity of HRRs across the population. A more cost-effective strategy for analyzing these regions would be to model each HRR by defining a representative repeat unit and estimating its approximate copy number, as demonstrated in *HRR modeling* approaches [20].

### Diploid versus consensus assemblies: genuine benefits versus practical reality

At present, it is difficult to justify generating HiFi reads, ultralong ONT reads, Hi-C data, parent-child trio data, and Bionano optical maps solely to produce a T2T diploid assembly of a non-human genome, when a near-complete T2T consensus assembly can be constructed from HiFi reads alone with minimal effort. That is why, the EBP recently projected that “*in the future, it may be possible to simplify sequencing so that high-quality genome assemblies can be generated from a single data type*” [10]. At present, since diploid assemblies remain expensive, achieving the EBP goal—generating 150,000 reference genomes representing half of known eukaryotic genera by 2030—is realistic only for haplotype-mixed assemblies.

One could argue that a T2T assembly of both haplomes would be important for analyzing variations within, for example, fungal or bird populations. However, such variations can also be captured by a *phased assembly* containing long contigs from both haplomes, which can be generated from HiFi reads alone [21–23]. Recent population-level sequencing studies of birds [24, 25] have adopted this strategy: generating only HiFi reads and assembling them into long phased contigs representing both haplomes. Such studies require a reference genome to establish a coordinate system for these phased contigs. In practice, a consensus assembly—effectively a “virtual haplome”—can serve this role as well as a true haplome. This consensus represents a “recombination” of the two parental haplotypes, producing a “virtual child” haplome that functions as a reference genome.

We illustrate the trade-off between diploid and consensus assemblies using the genome of the asexual, highly polymorphic (HP) leaf rust fungus *Puccinia triticina* (*Pt*). Because *Pt* fungi damage crops, they have been the focus of extensive sequencing efforts. However, short-read *Pt* sequencing projects produced fragmented assemblies, complicating functional annotation [26]. The first long-read consensus *Pt* assembly improved contiguity and greatly facilitated downstream analyses, enabling the prediction of genes, virulence factors, phylogenetic relationships, and other genomic features [27].

Because asexual fungi evolve through somatic mutations and haplotype exchanges, specific evolutionary questions arise regarding the history of these events across the fungal population. A T2T diploid assembly is better suited to address these questions. Duan et al., 2022 [14] generated such a T2T diploid *Pt* assembly using a combination of sequencing technologies and demonstrated its advantages over consensus assembly in addressing certain *Pt*-specific questions.

The asexual *Pt* fungus represents a rare case in which a diploid assembly of a non-human genome enables the studies of population demography in ways that the consensus assembly does not. Other applications of T2T diploid assemblies include the study of allele-specific gene expression and haplotype-specific combinations of disease-associated mutations [28]. However, these applications are either (i) limited to the human genome, (ii) require haplotypes of many genomes, which are rarely generated in non-human projects, or (iii) can be performed on phased assemblies almost as effectively as on complete haplotypes [29]. Thus, although a diploid assembly provides a more accurate representation of an individual genome, the advantages of such a costly assembly—relative to a haplotype-mixed assembly—are less pronounced in non-human studies.

### Early approaches to diploid assemblies

Early attempts to assemble HP genomes—those with more than 0.5% divergence between haplomes [30]—involved the generation of a consensus assembly, followed by the analysis of polymorphisms relative to this consensus [31–34]. This approach is contrasted by a more conservative one [35–39], in which segments from both haplomes are generated by enforcing strict criteria for overlapping reads; allelic relationships between haplotypes are reconstructed based on pairwise contig alignments.

However, these approaches—developed in the context of short-read sequencing data—resulted in highly fragmented assemblies. Subsequent long-read-based methods [21, 40–44] expanded on concepts originally developed for short-read diploid assemblies and resulted in more contiguous assemblies.

### Consensus assembly through the lens of genome decomposition

The consensus assembly problem shares similarities with the problem of inferring synteny blocks and blocks of segmental duplication (referred to as *blocks* below). The first block reconstruction algorithms were developed for constructing large human-mouse synteny blocks but were not designed for inference of blocks that account for segmental duplications [45–51]. Jiang et al., 2007 [52] used the concept of the *A-Bruijn graph* [53] for decomposing the human genome into the alphabet of blocks and revealing the mosaic structure of segmental duplications. Paten et al., 2008 [54], Pham and Pevzner, 2010 [55], and Minkin et al., 2013 [56] further described various *graph simplification* algorithms for inferring blocks in highly duplicated genomes.

Like consensus assembly algorithms, these graph simplification algorithms collapse bubbles in the de Bruijn graph (DBG) of a genome. However, eukaryotic genomes typically contain many regions which are not adequately represented by bubbles. Like the GenomeDecoder algorithm for genome decomposition [57], our Mosaic Genome Assembler (MGA) algorithm extends the set of operations used in block generation algorithms, representing the main improvement over previous approaches to consensus assembly. Another improvement is the use of the *multiplex de Bruijn graph* [22], which increases assembly contiguity and minimizes phase-switches.

### From overlap/string graphs to de Bruijn graph-based consensus assemblies

Early long-read approaches to generating a consensus assembly were based on the overlap/string graph paradigm rather than the de Bruijn graph paradigm. With the advent of long-read DBG assemblers, the T2T consortium has partially shifted from overlap/string graph assemblers, such as HiCanu [58] and hifiasm [21], to the DBG assembler Verkko [23], which has been recently used to assemble various mammalian genomes [7, 11–13, 59]. Because both DBG and overlap/string graph diploid assemblers are under active development [60, 61] and focus on integrating multiple sequencing technologies, their direct comparison remains a moving target. Nonetheless, as shown in Table 1 of [23], the phased DBG assemblers LJA [22] and Verkko [23] outperform overlap/string graph assemblers in HiFi-only assemblies.

Here, we introduce the MGA assembler that achieves higher contiguity than the state-of-the-art hifiasm assembler [21] while maintaining similar or better phase-switch rates. MGA generated near-complete consensus assemblies of all chromosomes in various fungal genomes and assemblies of multiple mammalian genomes with much higher contiguity than hifiasm.

## Results

### De Bruijn graphs

A path in a graph is *non-branching* if the indegree and outdegree of each internal node in this path are one. Herein, we use the term *DB graph* to refer to the *condensed DBG* that is obtained from the standard DBG by condensing all non-branching paths [22]. We refer to the DBG constructed on the set of *k*-mers of a string-set *Genome* as DB_*k*_(*Genome*) and to the DBG constructed on the set of *k*-mers of a read-set *Reads* as DB_*k*_(*Reads*). We refer to the “ground truth” haplomes of a diploid genome as Haplome_1_ and Haplome_2_. Unlike T2T diploid assembly, which seeks to reconstruct both Haplome_1_ and Haplome_2_, *phased assembly* aims to reconstruct the *phased genome graph* DB_*k*_(Haplome_1_ + Haplome_2_) by approximating it using the *phased DB graph* DB_*k*_(*Reads*) and generating contigs derived from this graph (Additional file 1: Supplementary Note 1). MGA uses the phased assembly graph generated by the LJA assembler [22] to generate a consensus assembly of these haplomes. We selected LJA as the engine for MGA because recent benchmarking (Table 1 in [23]) showed it to be the most contiguous and accurate HiFi-only assembler among the tested tools (Verkko, Flye, and hifiasm).

Given a read-set *Reads,* LJA performs error-correction on reads and constructs the graph LJA_*k*_(*Reads*) on the set of *k*-mers from these error-corrected reads that approximates the graph DB_*k*_(Haplome_1_ + Haplome_2_). Bankevich et al., 2022 [22] introduced the concept of the *multiplex de Bruijn* (*MDB*) *graph* multiDB_*k*_(*Reads*) with variable *k*-mer sizes that represents a more contiguous phased assembly: both LJA and Verkko generate MDB graphs. We use the term *assembly graph* to refer to either DB or MDB graphs. MGA generates a consensus assembly by simplifying the MDB graph, rather than the DB graph.

### Visualizing assembly graphs

We classify an edge in a graph as *short* if its length does not exceed a *length threshold* (default 20 kb) and as *long* otherwise. Assembly graphs often contain many short edges, which complicate their visualization. We construct a *contracted assembly graph* by contracting each short edge (*v*,*w*) into a new *contracted node* connected to all nodes adjacent to either *v* or *w*.

In the case when the diploid assembly of an *N*-chromosomal genome is available, we illustrate how individual chromosomes traverse the assembly graph using the *chromosome-colored graph* where each edge is colored into one of *N* colors. We align the sequence spelled by each edge in the assembly graph to each chromosome and assign each edge the color of the chromosome with the longest alignment span on this edge. Unless stated otherwise, we use minimap2 [62] with the “asm20” preset options (version 2.21-r1071) as the default aligner.

**Fig. 1:**
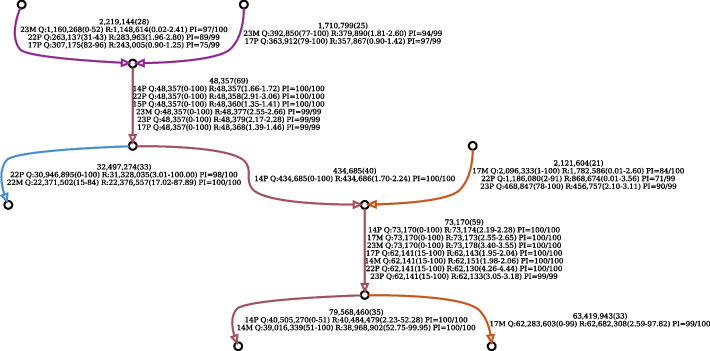
The largest component of the assembly graph MGA_5001_(BONOBO^*^) before launching the Deduplication module of MGA. This component includes edges representing assemblies of chromosomes 14, 17, and 22. Two vertical edges (lengths 48,357 bp and 73,170 bp) align to multiple chromosomes with full edge span (0–100) and PI_1_/PI_2_ = 100/100, indicating that these edges correspond to long nearly perfect repeats shared across chromosomes. We define a contig as a *near-complete consensus chromosome* if the merged span of its alignment on the maternal and paternal haplomes covers coordinates 1–99% of the corresponding haplome. Although chromosomes 14, 17, and 22 were not assembled into near-complete chromosomes, most of their sequences were captured in large contigs spanning 2.23–99.95% of chromosome 14, 2.59–97.82% of chromosome 17, and 3.01–100% of chromosome 22. If an edge aligns to multiple chromosomes, its color is defined by the chromosome with the largest span on the edge; ties are resolved first by the highest PI_1_ and then by the highest PI_2_. Since minimap2 occasionally splits a global alignment into multiple discontinuous local alignments [63], we merged local alignments from the same query to the same reference when the gap between them was less than 5% of the reference length. The alignment lengths were summed, and the PI_1_/PI_2_ of the merged alignment was computed as x/y, where x and y were obtained by proportionally weighting x_1_/y_1_ and x_2_/y_2_—the PI_1_/PI_2_ of the two alignments—according to their respective alignment lengths on the reference. The Figure was visualized using Graphviz v2.50.0 [64]

Figure 1 presents an example of a chromosome-colored assembly graph. Since LJA and several other HiFi assemblers operate on homopolymer-compressed reads, we assume that all read-sets analyzed below are homopolymer-compressed. Each edge is labeled with its length (bp) and read coverage (in parentheses) on the first line. Subsequent lines provide alignment information for the entire edge against the homopolymer-compressed haplomes: the chromosome ID (suffixes M/P denote the maternal and paternal haplomes); (Q:) the alignment length (bp) and coordinates (percentage) on the edge; (R:) the alignment length (bp) and coordinates (percentage) on the haplome; and the alignment percent identity reported as PI_1_/PI_2_. Here, PI_1_ is the standard percent identity defined as number of matches divided by the total number of matches, mismatches, insertions and deletions, and PI_2_ excludes gaps ≥ 10 bp. For example, the blue edge (length 32,497,274 bp, coverage 33 ×) in Fig. 1 aligns to the paternal chromosome 22 of the bonobo genome, “22P,” with PI_1_/PI_2_ = 98/100%. The alignment spans the entirety of the edge length (0–100%) and 3.01–100% of the length of chromosome 22P. This edge also aligns to maternal chromosome 22 with PI_1_/PI_2_ = 100/100%, spanning 15–84% of the edge length and 17.02–87.89% of chromosome 22M length.

### Outline of the MGA algorithm

**Fig. 2:**
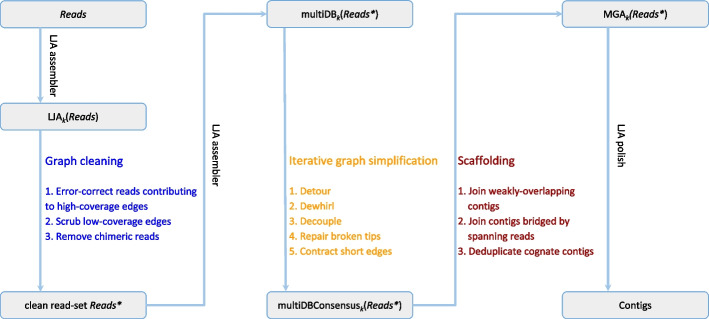
The MGA workflow. (i) Graph cleaning: MGA launches LJA to construct the graph LJA_*k*_(*Reads*). It uses the distribution of edge coverages to define the concepts of high-coverage edges (that require additional error-correction of reads traversing these edges) and low-coverage edges (that are typically false). Even though LJA includes error correction of reads, MGA performs an additional round of error correction—it corrects reads aligned to the high-coverage edges, scrubs reads contributing to low-coverage edges, and removes chimeric reads, resulting in a “clean” reads-set Reads^*^. MGA launches LJA again to construct the MDB graph multiDB_*k*_(Reads^*^). (ii) Iterative graph simplification: MGA iteratively performs operations of detouring, dewhirling, decoupling, repairing broken tips, and contracting short edges to generate the graph multiDBConsensus_*k*_(Reads^*^). (iii) Scaffolding: MGA connects contigs that were separated by the drops in coverage using either their short overlaps or reads spanning these contigs, producing an assembly graph with increased contiguity. It then identifies cognate contigs—those largely contained within another contig—and removes (deduplicates) them to avoid redundancy. This generates the final graph MGA_*k*_(Reads^*^), in which edges spell homopolymer-compressed contigs. MGA generates the final homopolymer-uncompressed contigs using LJA’s polishing procedure

While DBG assemblers primarily focus on generating phased HiFi assemblies, producing DBG-based consensus HiFi assemblies remains a poorly studied problem. MGA addresses this task by first launching LJA to error-correct the read-set *Reads* and to construct the graph LJA_*k*_(*Reads*) that represents the DB graph on the error-corrected reads. It generates the coverage distribution of edges in this graph and uses it to infer the *average coverage* (*Cov*) and the *low coverage* (*lowCov*) parameters that are used for defining the concepts of a *high-coverage edge* (coverage > 10 × *Cov*) and a *low-coverage edge* (coverage ≤ *lowCov*). Afterwards, MGA launches its graph cleaning, graph simplification, and scaffolding modules that we briefly describe below (Fig. 2).

#### Graph cleaning

Even though LJA constructs the DB graph on error-corrected reads, this graph contains false and chimeric edges [22]. Since many low-coverage edges result from incomplete error-correction in high-coverage regions, MGA additionally corrects reads mapped to such regions. It also removes all reads contributing to the identified low-coverage and chimeric edges, resulting in the clean read-set Reads^*^. Afterwards, it launches LJA to construct the MDB graph multiDB_*k*_(Reads^*^).

#### Graph simplification

**Fig. 3:**
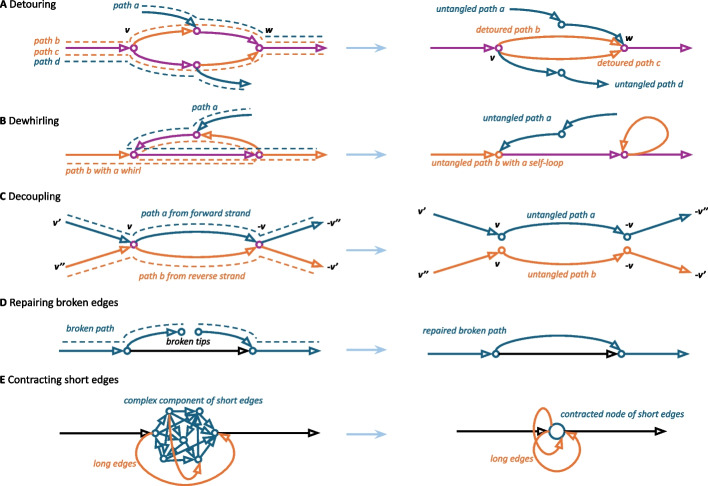
MGA graph simplification algorithm. Dashed paths show how the genome path traverses edges in the MDB graph. Blue and orange paths represent different paths in the MDB graph (shared edges between these paths are shown in purple). The graph multiDB_*k*_(Reads^*^) is iteratively simplified by performing: **A** Detouring: For two paths between nodes *v* and *w*, removing (detouring) one of the paths and replacing it with an edge from *v* to *w.* This panel illustrates two detouring operations: on the upper path of a bubble and on its bottom path; **B** Dewhirling: transforming a cycle (whirl) into a loop-edge that is later merged into a non-branching path representing a tandem repeat (not shown in the Figure); **C** Decoupling: separating forward and reverse strands tangled by reverse-complementary repeats resulting in genomic segments where both a *k*-mer *v* and its reverse complement (-*v*) occur on the same strand. The incoming edge (*v’*, *v*) is paired with the outgoing edge (-*v*, -*v’’*) and the incoming edge (*v’’*, *v*) is paired with the outgoing edge (-*v*, -*v’*); **D** Repairing broken tips: connecting broken tips induced by broken paths (typically occurring in low-coverage regions) in the graph; **E** Contracting short edges: contracting short edges into contracted nodes. Contracted nodes typically correspond to HRRs that are represented as HRR models in the MGA output. Long edges for which all incident edges are short will appear as long loops on the contracted nodes. Self-loops on the contracted nodes shorter than 1 Mb are incorporated into the node sequence as units of short tandem repeats (e.g., rDNA), whereas self-loops ≥ 1 Mb remain in the graph, as they are unlikely to represent single repeat units of short tandem repeats

We refer to a pair of parallel edges in a graph as a *simple bubble* and a pair of anti-parallel edges as a *simple whirl*. Most existing consensus assembly approaches simplify the assembly graph by iteratively collapsing simple bubbles, a procedure that removes one of the parallel edges in each bubble, with some assemblers also performing whirl-based simplifications. Similar to the short-read DBG-based approaches for diploid assembly of HP genomes [30, 65], MGA simplifies the DBG; however, it employs a more extensive set of operations (Fig. 3). It iteratively applies detouring, dewhirling, decoupling, broken tip repairing, and short edge contracting to transform the graph multiDB_*k*_(Reads^*^) into the graph multiDBConsensus_*k*_(Reads^*^).

#### Scaffolding

An edge (*v,w*) is *isolated* if it is the only edge connected to either *v* or *w*. It is classified as an *out-tip* if indegree(*w*) = 1 and outdegree(*w*) = 0 and as an *in-tip* if outdegree(*v*) = 1 and indegree(*v*) = 0. Two adjacent regions in the genome may be represented by a pair of isolated edges or an in-tip/out-tip pair (rather than a single edge) in the assembly graph due to a drop in read coverage at the border of these regions. The graph multiDBConsensus_*k*_(Reads^*^) often contains *weakly-overlapping* isolated edges/tips that spell contigs overlapping by less than *k* nucleotides. MGA identifies and reconnects weakly-overlapping edges and bridges short gaps between edges with *bridging read*s. For contigs spelled by all edges, MGA identifies *cognate contigs*—defined as shorter contigs whose sequences are aligned to a longer contig from a different haplome (referred to as an *essential contig*). It further performs *deduplication* by removing all cognate contigs, resulting in the final graph MGA(Reads^*^). MGA uncompresses homopolymer-compressed contigs as the final output using the polishing module of LJA.

### The consensus genome problem

Given a read-set *Reads* for a genome formed by haplomes Haplome_1_ and Haplome_2_, we define the *consensus genome* of Haplome_1_ and Haplome_2_ as a genome Haplome that maximizes sim(Haplome,Haplome_1_) + sim(Haplome,Haplome_2_), where sim(X,Y) is the score of the global alignment between genomes X and Y. As the first step towards solving the consensus assembly problem, we consider a simpler Consensus Genome (CG) Problem—constructing a consensus genome from the graph DB_*k*_(Haplome_1_ + Haplome_2_). We thus decompose the practical problem of constructing a consensus assembly into two parts: a simplified CG algorithm and its real-data adaptation, MGA, which assembles reads and accounts for complications such as sequencing errors, uneven genome coverage, etc. We emphasize that although the haplomes Haplome_1_ and Haplome_2_ are known, they are not available to the CG algorithm after the construction of the graph DB_*k*_(Haplome_1_ + Haplome_2_). We use the CG algorithm solely to illustrate various steps of MGA on a graph that is simpler to analyze.

### Datasets

To benchmark MGA, we used datasets from two fungi genomes (leaf rust fungus *Pt* and stripe rust fungus *Pst*) and four mammalian genomes (human, bonobo, giraffe, and sheep). We refer to T2T diploid assemblies of these genomes as *Genome*_RUST_, *Genome*_RUST-Pst_*, Genome*_BONOBO_, *Genome*_HUMAN,_ *Genome*_GIRAFFE_, and refer to the sheep paternal haplome assembly as *GenomeP*_SHEEP_. Information about these genomes is provided in Tables 1 and 2. Below we provide information about the corresponding read-sets (additional information is provided in Additional file 1: Supplementary Note 2).

**Table 1:** Statistics for fungi assemblies generated by various assemblers. MGA and hifiasm generated RUST and RUST-Pst assemblies with similar N50 values but MGA assembled more near-complete chromosomes and resulted in higher N90 values. MGA assembled all 18 chromosomes in the *Pt* genome into near-complete consensus chromosomes while hifiasm broke chromosome 17 of this genome into two long contigs and many short contigs. MGA assembled 19 long contigs for the RUST dataset; the additional long contig MGA assembled, of length 1.6 Mb, has 47% of its sequence mapped (percent identity 81%) to the bacterial genome *Herbaspirillum hiltneri* N3—which has been associated with wheat roots [66]. Chromosome 17 from the *Pt* genome was excluded from the phase-switch rate calculation because it contains a long rDNA repeat that evaded GENOME_RUST_ assembly, making phase-switch estimates unreliable (Additional file 1: Supplementary Note 4). Additional assessments of the consensus assembly are provided in Additional file 1: Supplementary Note 9. Hifiasm was run with option “--primary”. Falcon was run based on [67] (Additional file 1: Supplementary Note 3). Phase-switch rates were evaluated using GraphAligner v1.0.19 [68] (Additional file 1: Supplementary Note 4). The heterozygosity information was obtained from the papers published along with the datasets

**Assembler**	**#Contigs (essential contigs only)**	**#Long contigs** **(**≥ **1 Mb)**	**Total contig length** **(Mb)**	**N50 (Mb)**	**N90 (Mb)**	**Phase-switch rate**
**RUST**_**sim**_ (1 chromosome, haplome sizes 9.2/9.5 Mb, 59,704 HiFi reads (L50 = 13 kb, 29 × coverage))						
MGA	1	1	9.4	**9.4**	**9.4**	1.2%
LJA + purge_dups	14	6	18.5	3.0	0.9	1.0%
hifiasm	1	1	9.3	9.3	9.3	2.1%
HiCanu	10	6	18.1	2.9	0.9	**0.4%**
Falcon	12	5	12.1	2.1	0.7	11.1%
**RUST** (18 chromosomes, haplome sizes 123.9/121.6 Mb, 900,485 HiFi reads (L50 = 15 kb, 53 × coverage), heterozygosity 0.7%)						
MGA	39	19	128.2	7.5	**4.7**	4.0%
LJA + purge_dups	327	71	237.2	2.5	0.5	**2.6%**
hifiasm	136	19	133.6	**7.7**	2.5	3.7%
HiCanu	576	59	242.9	3.0	0.2	5.1%
Falcon	194	62	184.4	2.3	0.7	15.5%
**RUST-Pst** (18 chromosomes, haplome sizes 75.6/75.9 Mb, 705,557 HiFi reads (L50 = 14 kb, 62 × coverage), heterozygosity 0.3%)						
MGA	28	18	78.5	**4.7**	**2.9**	3.8%
LJA + purge_dups	863	22	143.1	0.6	0.0	**1.8%**
hifiasm	41	20	79.2	4.6	2.7	5.2%
HiCanu	392	22	132.5	2.6	0.1	4.4%
Falcon	86	24	81.8	2.9	0.9	15.8%

Larger N50 and N90 values and lower phase-switch rates are highlighted in bold

**Table 2:** Information about the BONOBO, HUMAN, GIRAFFE and SHEEP assemblies generated by MGA and hifiasm. Phase-switch rates were evaluated using the yak tool [21] by generating the *k*-mer spectra of the maternal and paternal haplomes and mimicking the *k*-mer analysis usually employed in genome assembly with trio binning [69]

Assembler	#Essential contigs	#Long contigs (≥ 1 Mb)	Total contig length (Mb)	N50 (Mb)	N90 (Mb)	Phase-switch rate
**BONOBO** (24 chromosomes, haplome sizes 3.1/3.2 Gb, N50 = 147.0 Mb, 14 million HiFi reads (L50 = 18 kb, 45 × coverage))						
MGA	80	58	3346	**108.5**	**45.8**	**2.3%**
hifiasm	152	95	3344	77.3	17.6	2.8%
**HUMAN** (23 chromosomes, haplome sizes 2.9/3.1 Gb, N50 = 146.8 Mb, 18 million HiFi reads (L50 = 17 kb, 48 × coverage))						
MGA	45	40	3,113	**143.4**	**66.8**	**0.6%**
hifiasm	127	65	3,078	95.4	36.5	0.9%
**GIRAFFE** (15 chromosomes, haplome sizes 2.4/2.6 Gb, N50 = 178.5 Mb, 13 million HiFi reads (L50 = 20 kb, 46 × coverage))						
MGA	38	23	2612	**167.0**	**71.5**	**0.4%**
hifiasm	100	36	2559	109.4	65.5	0.5%
**SHEEP** (27 chromosomes, paternal haplome size 3.0 Gb, N50 = 105.2 Mb, 10 million HiFi reads (L50 = 23 kb, 36 × coverage))						
MGA	78	44	3042	105.2	**61.3**	-
hifiasm	124	61	2965	**112.5**	61.2	-

Larger N50 and N90 values and lower Phase-switch rates are highlighted in bold

#### RUST dataset

The RUST dataset is formed by HiFi reads from the *Pt* genome [14]. Duan et al., 2022 [14] generated a hifiasm-based *Pt* assembly using HiFi reads, ONT reads, and Hi-C reads.

#### RUST-Pst dataset

The RUST-Pst dataset is formed by HiFi from *Pst* genome, a wheat pathogen [15]. To fix the deficiencies of previous fragmented *Pst* assemblies [70], Wang et al., 2024 [15] generated a hifiasm-based *Pst* assembly using HiFi reads and parent–child trio Illumina reads. This *Genome*_RUST-Pst_ assembly resulted in two haplomes with contig N50 of 4.17/4.60 Mb and 15/16 gapless chromosomes (out of 18 chromosomes in each haplome).

#### BONOBO dataset

The BONOBO dataset is formed by ≈14 million HiFi reads (L50 = 18 kb, 45 × coverage) from the bonobo genome. Yoo et al., 2025 [7] generated Verkko-based T2T bonobo assembly using HiFi reads, ultralong ONT, Hi-C, and parent–child trio Illumina reads.

#### HUMAN dataset

The HUMAN dataset is formed by ≈18 million HiFi reads (L50 = 17 kb, 48 × coverage) from the HG002 cell line. Jarvis et al., 2022 [71] generated Verkko-based T2T human assembly using HiFi reads and ultra-long ONT reads.

#### GIRAFFE dataset

The GIRAFFE dataset is formed by ≈13 million HiFi reads (L50 = 20 kb, 46 × coverage) from the Masai giraffe genome. Kim et al., 2025 [13] generated Verkko-based T2T giraffe assembly using HiFi reads, ONT duplex data, ultra-long ONT reads, and Hi-C data.

#### SHEEP dataset

The SHEEP dataset is formed by ≈10 million HiFi reads (L50 = 23 kb, 36 × coverage) from the Bighorn x Polypay sheep genome. Olagunju et al., 2025 [12] generated Verkko-based T2T assembly of the paternal haplome *GenomeP*_SHEEP_ using HiFi reads, ONT reads, ultra-long ONT reads, and Hi-C reads (the maternal haplome has not been assembled yet).

### Assembly tools

We benchmarked MGA, Falcon [41], HiCanu [58], Flye [72], hifiasm [21] and LJA + purge_dups (combining LJA with the purge_dups software for removing *haplotypic duplication* [73]). Additional file 1: Supplementary Note 3 provides information about these assemblers and the parameters used for benchmarking.

Our benchmarking primarily focused on the (LJA-based) MGA and hifiasm assemblers since a recent benchmarking study [74] identified LJA and hifiasm as the top-performing assemblers for HiFI reads. In our own benchmarking experiments, Flye produced inferior results and was therefore excluded from further analysis. This outcome is not surprising, as Flye was developed primarily for ONT rather than HiFi reads and was designed for phased rather than consensus assembly.

We evaluated each assembly using parameters N50, N90, and the *phase-switch rate* (Additional file 1: Supplementary Note 4). To eliminate bias from differences in internal deduplication strategies among assemblers, we applied a uniform standalone deduplication procedure that classifies a contig as cognate if it aligns across at least 90% of its length to a longer essential contig. We also evaluated MGA and hifiasm assemblies using the CRAQ tool [75] and the newly developed VirtualHaplome approach (described in Additional file 1: Supplementary Note 5), which uses QUAST-LG [76] to evaluate the virtual haplome aligned against a sparse DBG [77]. This analysis is described in Additional file 1: Supplementary Notes 6 and 7.

### Consensus haplome of human chromosome 21

To illustrate various steps of MGA, we show how its simpler version (the CG algorithm) works on the graph DB_*k*_(H21P + H21M) where the H21P and H21M represent the paternal and maternal haplomes of chromosome 21 of the HG002 human genome (38.9 Mb and 34.3 Mb, respectively).

The jumboDBG module of LJA constructed the graph DB_5001_(H21P + H21M) with 3,960/5,932 nodes/edges, 1,902 simple bubbles, and 36 simple whirls. Iterative processing of simple bubbles and simple whirls, followed by condensing non-branching paths, reduced the graph to 80 nodes and 112 edges (Additional file 1: Supplementary Note 8), demonstrating that these transformations are insufficient for producing contiguous consensus assemblies. However, detouring and dewhirling operations in MGA resulted in a single edge (and its reverse complement), representing the consensus assembly of chromosome 21 with a length of 39.8 Mb. This consensus assembly is 0.97 Mb longer than the longer haplome of chromosome 21; this is because the CG algorithm prioritizes the selection of longer edges while collapsing simple bubbles.

### Comparing MGA and hifiasm on a simulated read-set

Comparing a consensus assembler X with an assembler Y using haplomes generated by Y introduces a bias in favor of Y, because the Y-generated diploid assembly does not necessarily represent the ground truth [8]. To avoid such bias, we evaluated the assemblers using the RUST_sim_ dataset, which consists of simulated reads sampled from the RUST genome, rather than the real HiFi reads from the hifiasm-assembled RUST dataset. The RUST_sim_ dataset was generated by simulating HiFi reads from the two haplomes of the largest chromosome in *Genome*_RUST_ using pbsim3 v3.0.5 [78].

Table 1 shows that simple bubble collapsing in the phased LJA assembly using purge_dups produced an inferior consensus assembly, highlighting the need for developing MGA. This Table illustrates that MGA and hifiasm assembled the RUST_sim_ dataset into a single consensus contig, but MGA achieved a lower phase-switch rate than hifiasm (1.2% vs. 2.1%). In contrast, other assemblers produced from 10 to 14 essential contigs, indicating they are less suitable for consensus assembly. We therefore focus the following benchmarking on MGA and hifiasm.

### Assembly of the RUST dataset

LJA assembled the RUST dataset into a graph LJA_5001_(RUST) with 7,530/10,967 nodes/edges and 2,244 simple bubbles. Performing iterative bubble collapsing on this graph resulted in a *bubble-collapsed* graph with 2,998/4,169 nodes/edges and the contracted bubble-collapsed graph with 481/577 nodes/edges (Additional file 1: Supplementary Note 8 visualizes a small component of this graph). Most chromosomes in the *Pt* genome aggregate into the complex component with 272/364 nodes/edges (Additional file 1: Supplementary Note 8), illustrating that standard bubble collapsing is insufficient for generating contiguous consensus assemblies.

The graph cleaning module of MGA transformed the read-set RUST into a clean read-set RUST^*^ and produced the graph DB_5001_(RUST^*^) with 5,884/8,593 nodes/edges. Additional file 1: Table S1 summarizes the graph sizes at every stage of MGA. The graph DB_5001_(RUST^*^) and the graph DB_5001_(*Genome*_RUST_) (constructed from the two *Pt* haplomes and having 5,396/7,949 nodes/edges) have similar sizes, indicating that DB_5001_(RUST^*^) provides a reasonable approximation of DB_5001_(*Genome*_RUST_). MGA then transformed DB_5001_(RUST^*^) into multiDB_5001_(RUST^*^) with 1,411/2,014 nodes/edges and into the graph MGA_5001_(RUST^*^) with 126/78 nodes/edges. The 78 edges include 18 long isolated edges (and their reverse complements) spelling the consensus sequences (homopolymer-compressed) of 18 *Pt* chromosomes, representing a near-complete consensus assembly of the RUST dataset.

Long contigs (≥ 1 Mb) in MGA assemblies typically correspond to chromosomal contigs. Additional file 1: Supplementary Note 10 summarizes the annotation of the short contigs (< 1 Mb) using BlastN v2.16.0 + [79] and illustrates that they represent the mitochondrial *Pt* genome, segments of bacterial genomes associated with wheat roots, virus sequences, etc. [66, 80–83].

We constructed the consensus assemblies of the RUST dataset generated by Falcon, HiCanu, hifiasm, and LJA + purge_dups (Table 1). MGA and hifiasm were the only assemblers to generate a nearly ideal consensus assembly: MGA assembled all 18 *Pt* chromosomes into near-complete consensus contigs and hifiasm assembled 17 chromosomes into near-complete consensus contigs, but split chromosome 17 (that contains extra-long rDNA repeat) into two long contigs and many short contigs. Additional file 1: Supplementary Notes 8 and 11 provide detailed information about all MGA contigs that illustrate that MGA successfully assembled each chromosome (from near 0% to near 100% of either haplome). MGA and hifiasm had similar phase-switch rate (4.0% and 3.7%, respectively) but the phase-switch rate analysis for RUST and RUST-Pst may be biased in favor of hifiasm, as it was evaluated using the hifiasm-based diploid assemblies.

### Assembly of the RUST-Pst dataset

LJA assembled the RUST-Pst dataset into the graph LJA_5001_(RUST-Pst) containing 9,028/12,852 nodes/edges and 2,938 simple bulges. The iterative bubble collapsing on this graph resulted in the contracted bubble-collapsed graph with 362/364 nodes/edges. The largest component of the contracted graph comprises 78/102 nodes/edges (Additional file 1: Supplementary Note 8), illustrating the substantial complexity retained in the graph and the limitations of the standard bubble collapsing procedure.

The graph DB_5001_(RUST-Pst^*^) with 6,886/10,152 nodes/edges (Additional file 1: Table S1), showing similar complexity to DB_5001_(Genome_RUST-Pst_) with 6,142/9,078 nodes/edges. DB_5001_(RUST-Pst^*^) was transformed into multiDB_5001_(RUST-Pst^*^) with 1,658/2,622 nodes/edges and into MGA_5001_(RUST-Pst^*^) with 92/56 nodes/edges. 18 isolated edges in this graph spell 18 long consensus contigs, representing near-complete T2T consensus assemblies of the 18 *Pst* chromosomes (Additional file 1: Supplementary Notes 8 and 11).

We assembled the RUST-Pst dataset with Falcon, HiCanu, hifiasm, and LJA + purge_dup. Hifiasm generated single-contig assemblies for 16 out of 18 *Pst* chromosomes, split chromosomes 9 and 17 into several contigs (Additional file 1: Fig. S1), resulting in lower percent identity than MGA (Additional file 1: Supplementary Note 9), and a higher phase-switch rate than MGA (5.2% versus 3.8%). Thus, MGA improved on the hifiasm assembly even though the *Pst* haplomes were originally assembled by hifiasm in diploid mode, introducing a bias in favor of hifiasm.

Until recently, evaluating the quality of consensus assemblies was challenging because accurate diploid assemblies of both haplomes—needed as a reference—were unavailable. In the absence of a well-defined objective function for evaluating consensus assemblies and a QUAST-like tool for this purpose, previous quality assessment efforts were limited to metrics such as N50, N90, and BUSCO [84, 85]. Additional file 1: Supplementary Note 9 provides further analysis of MGA and hifiasm assemblies of RUST and RUST-Pst datasets.

### Assembly of the BONOBO dataset

LJA assembled the BONOBO dataset into the graph LJA_5001_(BONOBO) with 150,750/225,320 nodes/edges and 63,408 simple bubbles (69% of edges in this graph are short). The bubble-collapsed graph contained 23,932/35,044 nodes/edges and the bubble-collapsed contracted graph contained 5,589/8,192 nodes/edges. The largest connected component in the bubble-collapsed contracted graph contains 2,257/3,704 nodes/edges, highlighting the limitations of simple bubble collapsing. Additional file 1: Supplementary Note 8 presents a small component of this graph. MGA’s graph cleaning procedure resulted in the graph DB_5001_(BONOBO^*^) with 149,522/223,596 nodes/edges (Additional file 1: Table S1) that exhibits similar complexity to the graph DB_5001_(*Genome*_BONOBO_) with 132,096/197,984 nodes/edges. DB_5001_(BONOBO^*^) was transformed into multiDB_5001_(BONOBO^*^) with 9,160/15,382 nodes/edges and into MGA_5001_(BONOBO^*^) with only 294/160 nodes/edges, including ten isolated edges that represent the near-complete T2T consensus assemblies for ten chromosomes (Additional file 1: Supplementary Notes 8 and 11). Figure 1 visualizes the largest component of the graph MGA_5001_(BONOBO^*^) before the Deduplication module, where chromosomes 14, 17, and 22 are connected through long repeats. Deduplication further removed some edges in the component, making the resulting graph very simple.

**Fig. 4:**
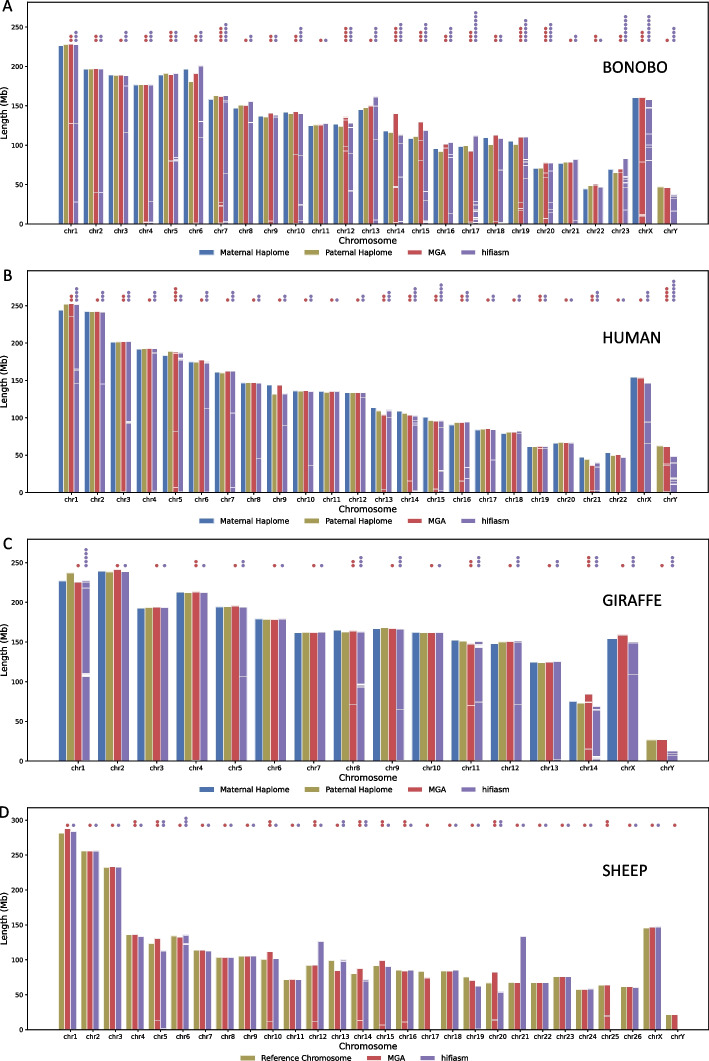
The lengths of MGA and hifiasm consensus contigs vs. true haplomes for the BONOBO (**A**), HUMAN (**B**), GIRAFFE (**C**), and SHEEP (**D**) datasets. Essential consensus contigs stacked within each bar were sorted according to the start/end coordinates of their alignments against the reference assembly. A contig aligned to haplomes from multiple chromosomes was assigned to the chromosome corresponding to the largest alignment span. The number of dots over each stacked bar indicates the number of long contigs (≥ 1 Mb) in this bar

MGA assembled the BONOBO dataset into 58 long contigs (with 16 contracted nodes representing HRR models) while hifiasm assembled it into 95 long contigs. MGA achieved a higher N50 than hifiasm (108.5 Mb vs. 77.3 Mb), and higher contiguity for 18 out of 25 chromosomes (Table 2 and Fig. 4). MGA assembled ten chromosomes (3, 6, 8, **9**, **11**, 13, 16, 18, **21**, and 23) into near-complete consensus contigs while hifiasm produced near-complete consensus contigs for three chromosomes shown in bold, and for chromosome 22 (Additional file 1: Supplementary Note 8).

Although hifiasm assembled chromosome 22 into a single contig (as compared to two contigs assembled by MGA), our analysis raises concerns about this assembly (Additional file 1: Supplementary Note 12). Bonobo chromosomes 22 and 14 share a perfect 23,544 bp repeat—spanned by no HiFi reads—making it impossible to unambiguously bridge this region. Thus, MGA assembly of chromosome 22 into 2 contigs may represent a more adequate attempt at assembling this chromosome.

Below we briefly describe assemblies of the HUMAN, GIRAFFE, and SHEEP datasets. Additional file 1: Supplementary Notes 8 and 11, as well as Table 2, provide detailed information about these assemblies.

### Assembly of the HUMAN dataset

LJA assembled the HUMAN dataset into the graph LJA_5001_(HUMAN) with 240,594/360,404 nodes/edges and transformed it into multiDB_5001_(HUMAN^*^) with 31,276/56,652 nodes/edges. The final graph MGA_5001_(HUMAN^*^) contained only 154/92 nodes/edges, including 14 edges that represent the near-complete consensus assemblies for 14 out of 23 human chromosomes.

MGA assembled the HUMAN dataset into 40 long contigs with N50 = 143.4 Mb (with 11 contracted nodes representing HRR models) while hifiasm assembled it into 65 long contigs with N50 = 95.4 Mb (Fig. 4). MGA assembled 14 chromosomes (2, 4, 6, 7, 8, 9, 10, **11**, 12, 17, **18**, **20**, and X) into near-complete consensus contigs, while hifiasm assembled 3 chromosomes (shown in bold) into near-complete consensus contigs.

### Assembly of the GIRAFFE dataset

LJA assembled the GIRAFFE dataset into the graph LJA_5001_(GIRAFFE) with 194,026/290,648 nodes/edges and transformed it into multiDB_5001_(GIRAFFE^*^) with 17,112/28,666 nodes/edges. The final graph MGA_5001_(GIRAFFE^*^) contained only 142/76 nodes/edges, including 12 edges that represent the near-complete consensus assemblies for 12 out of 15 giraffe chromosomes. Each of the other chromosomes was assembled into only 2–3 contigs.

MGA assembled the GIRAFFE dataset into 23 long contigs (with 15 contracted nodes representing HRR models) while hifiasm assembled it into 36 long contigs. MGA achieved higher N50 than hifiasm (167.0 Mb vs. 109.4 Mb), and higher or similar contiguity for each chromosome (Fig. 4). The total length of chromosome Y assembled by hifiasm is significantly smaller than that of the true haplome Y, indicating that hifiasm may have missed some segments of this chromosome.

MGA assembled 12 chromosomes (1, **2**, **3**, **4**, 5, **6**, **7**, 9, **10**, 12, **13**, and X) into near-complete consensus contigs, while hifiasm assembled 7 chromosomes (shown in bold above) into near-complete consensus contigs. MGA assembled one more contig than hifiasm for chromosome 4. One of the two contigs assembled by MGA for this chromosome is a near-complete assembly, and another represents a highly heterozygous region that was not deduplicated due to high divergence.

### Assembly of the SHEEP dataset

LJA assembled the SHEEP dataset into the graph LJA_5001_(SHEEP) with 14,640/21,384 nodes/edges and transformed it into multiDB_5001_(SHEEP^*^) with 3,390/4,974 nodes/edges. The final graph MGA_5001_(SHEEP^*^) contained only 262/156 nodes/edges, including 20 edges that represent the near-complete consensus assemblies for 20 out of 27 sheep chromosomes.

MGA assembled the SHEEP dataset into 44 long contigs (with 29 contracted nodes representing HRR models) while hifiasm assembled it into 61 long contigs. For most chromosomes, MGA and hifiasm generated a similar number of contigs. MGA generated more long contigs for chromosomes 4, 10, 12, 15, and 16, but for each of these chromosomes (except for chromosome 16), at least one long contig assembled by MGA represents a near-complete assembly; MGA assembled two contigs for these chromosomes because diverged contigs from highly heterozygous regions were not deduplicated. MGA achieved slightly lower N50 than hifiasm, but the N50 obtained by hifiasm was larger than that of the reference genome, suggesting that hifiasm misassembled some chromosomes. Our analysis revealed that hifiasm assembled two long chimeric contigs and missed the chromosome Y (Fig. 4), indicating that it made risky decisions in its assembly process.

MGA assembled 20 chromosomes (1, **2**, **3**, 4, 6, 7, **8**, **9**, **10**, **11**, **15**, **18**, 20, 21, **22**, **23**, **24**, **26**, **X**, and Y) into near-complete consensus contigs, while hifiasm assembled 13 chromosomes (shown in bold) into near-complete consensus contigs (Fig. 4).

### Running time and memory footprint

Table 3 provides information about the runtime and memory footprint for MGA and hifiasm on mammalian datasets. Additional file 1: Table S2 provides information about the runtime and memory footprint for all analyzed assemblers on the RUST dataset.

**Table 3:** The running time and memory footprint for MGA and hifiasm on the mammalian datasets. All assemblers were run using default parameters for assembling HiFi reads and the thread numbers were set to 50. Hifiasm was run with option “--primary”

Read-set	HUMAN		BONOBO		SHEEP		GIRAFFE	
Assembler	**MGA**	**hifiasm**	**MGA**	**hifiasm**	**MGA**	**hifiasm**	**MGA**	**hifiasm**
System time (seconds)	37591	20922	63406	10553	82483	35495	23087	17491
User time (seconds)	2321586	2893217	4127372	1760970	17033541	5004092	1592134	4418503
Elapsed time (h:mm:ss)	31:37:28	20:40:26	48:07:44	12:52:40	158:15:06	31:57:07	28:47:57	28:52:53
Memory (Mb)	206528	393431	272567	255099	420709	221298	203422	292322

Recent benchmarking of LJA and hifiasm highlighted a tradeoff: LJA achieves a higher assembly contiguity, while hifiasm offers a faster runtime [86]. Because LJA—like other DBG-based HiFi assemblers—is slower than hifiasm, and because the runtime of MGA is dominated by LJA itself, MGA is slower than hifiasm. Consequently, the choice between MGA and hifiasm for consensus assembly reflects a broader tradeoff between assembly quality and computational efficiency. We note that the runtime of a consensus assembly is still a small fraction of the runtime of a diploid assembly that uses multiple sequencing technologies.

## Discussion

We have presented the MGA algorithm for constructing haplotype-mixed assemblies of diploid genomes. Even though MGA uses the DB graph generated by the LJA assembler [22], it could also be modified to use DB graphs generated by other DBG assemblers such as Verkko [23] and mdBG [87].

MGA extends the bubble collapsing approach and complements it with various heuristics. Interestingly, the long-read assemblers Flye [72] and NextDenovo [88], initially designed for assembling long error-prone reads, implicitly generate consensus assemblies of highly similar regions in two haplomes, e.g., regions that are more than 99% similar. Even though these consensus assemblies can be further improved by post-processing that generates *haplotigs* [73], we showed that simple bubble collapsing is still insufficient for constructing contiguous consensus assemblies.

Diploid assemblies offer certain advantages over consensus assemblies but their high cost can be difficult to justify in some sequencing efforts, such as the 1000 + Fungal Genomes Project [89] or the Fungal Genome Initiative [90]. This expense explains why some large sequencing consortia, such as the EBG Project, do not prioritize diploid assemblies [9]. The T2T consensus assemblies, such as the *Pt* and *Pst* fungal assemblies generated by MGA, provide a cost-effective alternative to diploid assemblies. The utility of such assemblies is well-documented—for example, the consensus assemblies of fungal genomes enabled many downstream applications and provided various biological insights [91].

MGA assemblies may fail to provide the contiguous sequence of each chromosome—for example, when both haplomes in multiple chromosomes share a long perfect repeat that is not bridged by any HiFi read. However, if the goal is to generate an inexpensive T2T consensus assembly in such cases, one can generate a small set of ONT reads with very low coverage to bridge long repeats and coverage gaps. Alternatively, one can use the T2T assembly of a similar species to resolve repeats and close coverage gaps using reference-assisted assemblers [92–94]. For example, since human and bonobo genomes are nearly collinear and since rearrangement breakpoints are not coordinated with drops in read coverage, using the human genome for a reference-assisted consensus assembly of the bonobo genome would resolve all repeats and close all coverage gaps. We note that current reference-assisted assemblers, which work well for consensus assemblies, are not suitable for diploid assemblies.

## Conclusions

MGA addresses the challenge of generating contiguous and accurate haplotype-mixed assemblies. Our benchmarking demonstrates that it substantially improves upon state-of-the-art haplotype-mixed assembly tool hifiasm.

One might argue that with continued increases in HiFi/ONT read length and accuracy, diploid assembly will eventually become routine using a single sequencing technology. However, that moment has not arrived. Current diploid assemblies still require multiple technologies and remain time-consuming, semi-automated endeavors. Moreover, even dramatic improvements in read length—say, if HiFi reads doubled in length tomorrow—would not eliminate this challenge. The fundamental obstacle to complete diploid assembly is that homologous chromosomes typically share long identical regions, which cannot be resolved even with very long reads. In contrast, haplotype-mixed assemblies benefit directly from increased read length because each individual haplome contains relatively few long perfect repeats. Thus, the contiguity of haplotype-mixed assemblies improves rapidly with longer reads, while diploid assemblies remain fundamentally limited by inter-haplome similarity.

## Methods

### Consensus genome problem

Previous studies have not provided an algorithmic formulation of the Consensus Assembly (CA) Problem, hindering the benchmarking of consensus assembly methods due to the absence of a rigorous objective function. Here, we introduce a deliberately oversimplified formulation of the CA Problem—the Consensus Genome (CG) Problem—that provides an intuitive heuristic framework for consensus assembly and motivates the development of the MGA algorithm.

For simplicity, we formulate the CG problem using the graph DB_*k*_(Haplome_1_ + Haplome_2_) of two unknown circular haplomes Haplome_1_ and Haplome_2_, rather than the graph DB_*k*_(*Reads*). The objective is to generate a *haplome-pair*—two virtual circular haplomes, X_1_ and X_2_, that result in the same graph DB_*k*_(X_1_ + X_2_) as DB_*k*_(Haplome_1_ + Haplome_2_). Either X_1_ or X_2_ can serve as a consensus genome, but ideally, X_1_ and X_2_ should be similar to each other to reflect the concept of a consensus genome. The similarity sim(X_1_,X_2_) between circular strings X_1_ and X_2_ is defined as the score of their highest-scoring global alignment.

It may appear that the described problem does not adequately capture the spirit of genome assembly since it does not penalize assembly errors—for example, rearrangements in X_1_ and X_2_ as compared to Haplome_1_ and Haplome_2_. We note that Haplome_1_ and Haplome_2_ are unknown and that our real goal is to construct the graphs DB_*k*_(X_1_) and DB_*k*_(X_2_) (rather than strings X_1_ and X_2_) and output contigs in these graphs. In this framework, even if X_1_ and X_2_ represent rearranged versions of the unknown Haplome_1_ and Haplome_2_, our hope is that Haplome_1_ and Haplome_2_ represent traversals of DB_*k*_(X_1_) and DB_*k*_(X_2_) and thus can be represented by a set of contigs in DB_*k*_(X_1_) and DB_*k*_(X_2_), respectively.

Given an edge (*v,w*) in the assembly graph of a *Genome*, we define seq(*v,w*) as the string spelled by this edge and mult(*v,w*) as its *multiplicity*—the number of times the string seq(*v,w*) occurs in *Genome*. We represent each edge (*v,w*) in a multigraph as mult(*v,w*) parallel edges. A graph is called *2-Eulerian* if it contains two non-empty cycles such that each edge in the graph belongs to exactly one of these cycles. The graph DB_*k*_(Haplome_1_ + Haplome_2_) is 2-Eulerian (each edge in this graph belongs to exactly one of two circular haplomes) and *balanced* (the outdegree of each node equals its indegree). A cycle in a connected graph is called *binding* if the removal of its edges does not disconnect the graph. Each binding cycle spells a virtual haplome X_1_ and removing this cycle leaves the graph connected and balanced. Each Eulerian cycle in the resulting graph defines a virtual haplome X_2_ that forms a haplome-pair with the haplome X_1_.

The objective of the CG Problem is to decompose the graph DB_*k*_(Haplome_1_ + Haplome_2_) into a haplome-pair (X_1_,X_2_) such that DB_*k*_(X_1_ + X_2_) = DB_*k*_(Haplome_1_ + Haplome_2_) and sim(X_1_,X_2_) is maximized among all possible haplome-pairs (as explained earlier, we output DB_*k*_(X_1_) and DB_*k*_(X_2_) rather than X_1_ and X_2_). Even though the existing consensus assembly algorithms (including MGA) focus on generating a single virtual haplome, they can often be adapted to generate a haplome-pair. For example, a simple bubble-collapsing algorithm can yield one virtual haplome by removing the shorter edge of each bubble and another virtual haplome by removing the longer edge. Although we defined the CG Problem for circular strings, it generalizes naturally to linear strings.

Below we describe the MGA algorithm that can be viewed as a heuristic for solving the CG Problem.

### Path removal

Given a path *P* = *P*(*v*,*w*) between nodes *v* and *w* in an assembly graph, we define its multiplicity mult(*P*) as the smallest multiplicity of its edges and denote the sequence spelled by this path as seq(*P*). A path is *simple* if it visits each of its internal nodes exactly once (a cycle that visits each of its nodes once is a special type of a simple path). A path is *short* if it has at most *maxPathSize* (default 8) edges.

Given a simple path *P* = *P*(*v*,*w*) in an assembly graph *G*, the *path removal operation* PathRemoval(*G*,*P*) (i) reduces multiplicities of all edges in *P* by mult(*P*) with follow-up removal of its zero-multiplicity edges, and (ii) adds a new edge (*v, w*) with mult(*v,w*) = mult(*P*) and seq(*v,w*) = seq(*P*). A path removal is called *binding* if it does not increase the number of weakly-connected components or tips in the graph. In a special case when a path *P* is a cycle that starts and ends at a node *v*, the step (ii) of PathRemoval(*G*,*P*) adds a loop-edge (*v,v*) at node *v* with mult(*v,v*) = mult(*P*) and seq(*v,v*) = seq(*P*).

### Detours

Two simple paths *P* = (*v*,*w*) and *P’* = *P’*(*v*,*w*) in a graph form a *detour Detour* = (*P*,*P’*) if they share no other nodes but their starting and ending nodes *v* and *w*. The percent identity of the detour is defined as the percent identity between seq(*P*) and seq(*P’*). A simple bubble, formed by two parallel edges, represents the simplest example of a detour.

A path in an assembly graph is *unambiguou*s if either (i) all its internal nodes have outdegree 1 or (ii) all its internal nodes have indegree 1. The concept of an unambiguous path generalizes the concept of a non-branching path since it also spells a contiguous segment of the genome.

If both paths *P* and *P’* in a detour are unambiguous, the *detouring* operation performs PathRemoval(*G*,*P*) and PathRemoval(*G*,*P’*), resulting in a simple bubble that is further collapsed by removing its shorter edge and adding its multiplicity to the longer edge. If *P* is an unambiguous path and *P’* is not an unambiguous path in a detour *Detour* = (*P*,*P’*), we define the *detouring operation* Detouring(*G*,*Detour*) as the result of (i) reducing multiplicities of all edges in *P* by mult(*P*) with follow-up removal of its zero-multiplicity edges, and (ii) adding mult(*P*) to the multiplicities of all edges in *P’*.

A detour *Detour* = (*P*,*P’*) is *short* if both its paths are short. A short binding detour *Detour* = (*P*,*P’*) is *valid* if the percent identity between *P* and *P’* exceeds the percent identity threshold *minPI* (default 60%).

### Whirls

A detour that starts and ends at the same node *v* forms a special type of a detour called a *whirl*. One path of such a detour is denoted as *Whirl* = *Whirl*(*v*) and the other path is an empty path from node *v*. We denote the sequence spelled by a single traversal of *Whirl* as seq(*Whirl*) and note that a whirl traversed multiple times represents a tandem repeat. Given a multi-edge whirl *Whirl* = *Whirl*(*v*) in a graph *G*, we define the *dewhirling operation* Dewhirling(*G*,*Whirl*) as the result of PathRemoval(*G*,*Whirl*), which generates a loop-edge at node *v*. We classify a whirl as *unambiguous* if it represents an unambiguous path. An example of an unambiguous whirl is given by a *simple whirl v*_*1*_*v*_*2*_*v*_*1*_ formed by anti-parallel edges (*v*_*1*_*,v*_*2*_) and (*v*_*2*_*,v*_*1*_), where (*v*_*2*_*,v*_*1*_) is the only outgoing edge from *v*_*2*_*.* We classify an unambiguous binding whirl as a *valid whirl.*

### Greedy heuristic for the consensus genome problem

A natural heuristic for solving the CG Problem proceeds as follows: (i) Find a binding detour formed by the two most similar paths in the graph, perform a detouring operation on this detour, and iterate until the resulting graph has no detours. (ii) Identify a shortest binding whirl in the graph, perform a dewhirling operation on this whirl, and iterate until the resulting graph has no whirls. (iii) Select an arbitrary binding cycle in the resulting graph, and label its sequence as a haplome X_1_, remove this cycle from the graph and select an arbitrary Eulerian path in the remaining graph as an alternative haplome X_2_.

MGA constrains this heuristic to valid detours and whirls and complements it with additional heuristics. Importantly, graph simplification operations of MGA work with the MDB graph rather than the DB graph, which improves the contiguity of the consensus assembly and reduces the phase-switch rate. We first describe the CG algorithm for constructing a consensus genome from the graph DB_*k*_(Haplome_1_ + Haplome_2_) with known multiplicities of edges and later modify it into the more complex MGA algorithm for generating a consensus assembly from the graph DB_*k*_(*Reads*) with unknown multiplicities but known read coverages of edges.

The CG algorithm (Additional file 1: Supplementary Note 13) iteratively performs detouring and dewhirling on valid detours and whirls in the graph *Graph* = DB_*k*_(Haplome_1_ + Haplome_2_). Instead of returning a haplome-pair (like in the abstract CG Problem), it returns the graph after all valid detouring and dewhirling operations—the contigs in the graph represent the consensus genome derived from the phased DB graph of two unknown haplomes.

### From CG algorithm to MGA algorithm

The CG Problem represents an abstraction that differs from the real challenge of consensus assembly in a few ways. Below, we describe these differences and how MGA addresses them.

- (i) Since the graph of haplomes DB_*k*_(Haplome_1_ + Haplome_2_) is unknown, MGA uses the assembly graph of reads LJA_*k*_(*Reads*) that approximates this graph. Although LJA already performs error correction, we found that these corrections are incomplete in regions of high coverage (Additional file 1: Fig. S2). Since even a small fraction of uncorrected reads leads to a deterioration of the consensus assembly, MGA improves error-correction in LJA: it corrects reads in high-coverage regions and removes reads that contribute to low-coverage and chimeric edges. These modules produce a clean read-set Reads^*^ and the clean MDB graph multiDB_*k*_(Reads^*^).
- (ii) Since the multiplicities of edges in DB_*k*_(Reads^*^) are not known, MGA approximates them using the read coverage of edges in this graph and further extends the coverage information to multiDB_*k*_(Reads^*^).
- (iii) Since the MGA attempts to solve a more complex problem than the CG Problem, it needs to be generalized to handle direct and reverse-complementary strands and complex components consisting of short edges that represent repetitive regions (Fig. 3). MGA introduces additional procedures to address these complications, such as strand decoupling and contracting short edges.
- (iv) The CG Problem is defined for a 2-Eulerian graph DB_k_(Haplome_1_ + Haplome_2_), but the graph multiDB_*k*_(Reads^*^) is not necessarily 2-Eulerian. This discrepancy arises from the presence of multiple chromosomes, false edges, breaks in coverage by reads, and challenges in inferring accurate multiplicities from read coverages. MGA introduces various procedures to address these issues, including repairing broken tips, scaffolding, and deduplication.

**Fig. 5:**
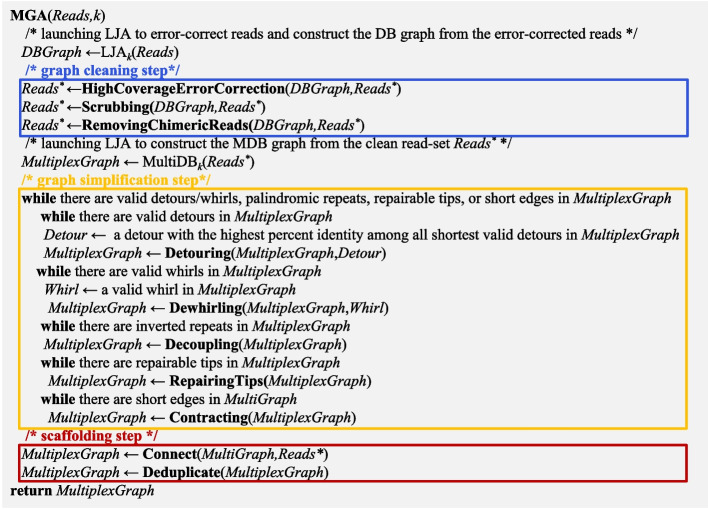
The pseudocode of the MGA algorithm. Blue, orange, and magenta sections represent the graph cleaning, graph simplification, and scaffolding steps of MGA, respectively

Figure 5 presents the pseudocode of the MGA algorithm. Below we describe all functions used in the MGA algorithm.

### Inferring edge multiplicities

Given an edge (*v,w*) in the assembly graph, we define cov(*v,w*) as its coverage by reads. LJA defines the coverage of an edge as the average coverage of all (*k* + 1)-mers in this edge, rather than the average coverage of all positions in this edge.

The graph LJA_*k*_(*Reads*) does not provide information about the multiplicities of its edges (the number of times the edge is traversed by the unknown haplomes). We approximate the multiplicity of an edge by dividing its read coverage by the estimated read coverage of a haplome (i.e., the average read coverage *Cov*) and rounding this estimate to the closest integer. The parameter *Cov* is computed by identifying the maximum in the histogram of edge coverages (Additional file 1: Fig. S3). Since LJA does not compute edge coverages in the MDB graph, MGA has an additional module to compute them (Additional file 1: Supplementary Note 14).

MGA recomputes the read coverage during each graph transformation and uses it in place of multiplicity in the detouring and dewhirling operations. Edge multiplicities are inferred from read coverages only when necessary. For example, we estimate the multiplicity mult(*Whirl*) of a whirl (the number of times it is traversed by the genome) by the minimum estimated multiplicities of its edges. In the case when |seq(*Whirl*)|× mult(*Whirl*) is smaller than the average read length, this estimate is further improved by checking if there exists a read that starts (ends) before (after) traversing *Whirl*. If such a read exists, mult(*W*) is estimated as the number of times this read traverses *Whirl*.

### Correcting reads that contribute to high-coverage edges in the de Bruijn graph

The large number of reads contributing to a high-coverage edge triggers mistakes in the LJA error correction procedure and leads to artifacts in the constructed assemblies (Additional file 1: Fig. S2). MGA estimates the average read coverage (*Cov*) edge coverage distribution in the graph of LJA_*k*_(*Reads*) (Additional file 1: Fig. S3) and classifies edges with coverage at least 10 × *Cov* as high-coverage edges. We define the *high-coverage DB graph* LJA_*k*_^+^(*Reads*) by retaining only high-coverage edges in LJA_*k*_(*Reads*) and condensing the non-branching paths in the resulting graph. Even though the graph LJA_*k*_^+^(RUST) is relatively small (15 edges of total length 87,046, without counting reverse complements), LJA fails to correct reads aligned to this graph, resulting in an explosion of erroneous edges in LJA_*k*_(RUST).

To correct reads contributing to high-coverage edges, MGA aligns reads to edges of LJA_*k*_^+^(*Reads*) using minimap2 with the map-hifi option. Aligned reads are corrected using the corresponding segment of the aligned edge—from the alignment start to end coordinates—if the alignment has > 99% percent identity and spans > 95% of the read length. If a read is aligned to multiple edges in LJA_*k*_^+^(RUST), the alignment with the longest span on the read is selected for correction. Given a DB graph *G* and a read-set *Reads*, we refer to the procedure of correcting reads contributing to high-coverage edges in *G* as a function HighCoverageErrorCorrection(*G*,*Reads*).

### Scrubbing low-coverage edges

Even though the graph DB_k_(*Reads*) approximates DB_*k*_(Haplome_1_ + Haplome_2_), there are many differences. The most common difference is the presence of low-coverage edges in DB_*k*_(*Reads*) that represent artifacts of the error-correction step in LJA and are not present in DB_*k*_(Haplome_1_ + Haplome_2_). Additional file 1: Fig. S3 shows the histogram of edge coverages in LJA_5001_(RUST) and reveals three peaks in this histogram: peak 0 at ~ 4 × coverage, corresponding to low-coverage (typically false) edges; peak 1 at ~ 27 × coverage (*Cov*), corresponding to edges with multiplicity 1; and peak 2 at ~ 56 × coverage, corresponding to edges with multiplicity 2. The dip between peaks 0 and 1, located at ~ 10 × coverage (*lowCov*), marks the boundary between true and false edges, with the vast majority of edges with coverage below *lowCov* being false. Given the threshold *lowCov*, the *scrubbing* operation Scrubbing(*G,Reads*) removes all reads contributing to low-coverage edges (with coverage less than *lowCov*).

### Removing chimeric reads

*Chimeric edges* in the assembly graph are caused by *chimeric reads* that “connect” different regions of the genome, i.e., represent a concatenated *prefix*-*suffix* where substrings *prefix* and *suffix* do not immediately follow each other in the genome. Even though long-read assemblers try to identify and remove chimeric reads, our analysis revealed that they fail to remove some chimeric reads, especially the rather common *endpoint chimeric reads* that connect a segment from the start/end of one chromosome to a segment from another chromosome.

To identify chimeric edges, we use the concept of *edge support* defined as follows: a read supports the edge (*v,w*) in the assembly graph if its alignment to this graph (i) starts in node *v* or before node *v*, and (ii) ends on edge (*v,w*) after node *v*. Since chimeric edges typically have very few reads supporting them, an edge is classified as chimeric if there are fewer than *chimera* reads supporting it (default value *chimera* = 2). Given an assembly graph *G* and the read-set *Reads*, the *chimeric edge removal operation* RemovingChimericReads(*G,Reads*) identifies chimeric edges in *G* and removes the chimeric reads supporting these edges.

### Decoupling forward and reverse strands

When the forward and reverse strands share long repeats (referred to as *inverted repeats*), the MDB graph *G* contains connected components that represent both forward and reverse strands. MGA attempts to simplify such components using the function Decoupling(*G*) that iteratively decouples inverted repeats.

An edge (*v,w*) is defined as a *2-in-2-out edge* if indegree(*v*) = outdegree(*w*) = 2 and outdegree(*v*) = indegree(*w*) = 1. We denote the two incoming edges into *v* as (*v’*,*v*) and (*v’’*,*v*) and the two outgoing edges from *w* as (*w*,*w’*) and (*w*,*w’’*). A 2-in-2-out edge (*v,w*) is classified as an inverted repeat if *w, w’,* and *w’’* are reverse complementary to *v*, *v’*, and *v’’*, respectively. Given an inverted repeat (*v, w*) in a graph *G*, the *decoupling operation* transforms the graph as follows (Fig. 3C): (i) adds a new edge (*v’, w’’*) with the sequence and multiplicity of the path *v’,v,w,w’’*; (ii) adds a new edge (*v’’,w’*) with the sequence and multiplicity of the path *v’’,v,w,w’*; (iii) removes edges (*v’*,*v*), (*v’’*,*v*), (*v,w*), (*w*,*w’*) and (*w*,*w’’*) from *G*.

### Repairing broken tips

Additional file 1: Fig. S4 illustrates an example of an edge that is preserved in one haplome but broken into an out-tip and an in-tip in another haplome. Frequent haplome-specific coverage breaks result in broken edges and typically require ultralong ONT reads to repair these breaks in diploid assemblies. However, coordinated edge-breaks in both haplomes (e.g., coverage breaks in both edges of a bubble) are rare. Therefore, a consensus assembly can often be constructed without ultralong ONT reads by using the unbroken edge in one haplome to “repair” the broken edge in the other haplome.

For each out-tip (*v,w*), MGA defines the *RepairEdge*(*v,w*) as the edge (*v,u*) (*u* ≠ *w*) that maximizes the prefix alignment score between seq(*v*,*w*) and seq(*v,u*) among all outgoing edges from *v*. A *RepairEdge*(*v*,*w*) is considered *valid* if the percent identity of this alignment exceeds the threshold *minRepairPI* (default 80%). For each in-tip (*u*,*t*), *RepairEdge*(*u,t*) is defined similarly.

MGA classifies an out-tip (*v*,*w*) and an in-tip (*u*,*t*) as a *tip-pair* if they have the same repair-edge (*v,t*). Given such a tip-pair in an MDB graph, the *repairing operation* removes tips (*v*,*w*) and (*u*,*t*) and increases the coverage of (*v,t*) by the average coverage of the tips (*v*,*w*) and (*u*,*t*). MGA further extends the repairing operation to all out-tips and in-tips that are not in tip-pairs. Such tips often represent a special case of a broken edge, e.g., when an out-tip (*v*,*w*) is present in the graph, but an in-tip (*u*,*t*) is not. Given the out-tip (*v,w*) in a MDB graph *G*, the repairing operation identifies *RepairEdge*(*v,w*) as described above, increases its coverage by cov(*v,w*), and removes the out-tip (*v,w*) (in-tips are repaired similarly). Additional file 1: Supplementary Note 15 describes a more general operation that extends repairing broken edges to repairing broken paths. The function RepairingTips(*G*) iteratively repairs all broken edges and paths in the MDB graph *G*.

### Contracting short edges

Some difficult-to-assemble HRRs may contain errors even in the most advanced T2T projects [8]. MGA uses HRR models [20] rather than accurate HRR sequences when it is unclear how to resolve an HRRs using HiFi reads. Diploid T2T assemblies use HRR models only for the most complex HRRs and typically resolve more HRRs than consensus T2T assemblies, highlighting their advantage over consensus assemblies in HRR-focused studies.

Because HRRs are typically built from relatively short repetitive units, MGA iteratively contracts all short edges in the MDB graph *G* (function Contracting(*G*)). If this operation introduces a non-short loop to a contracted node, it indicates the loop is a long edge that was previously connected to only short edges before contraction. When the sequence spelled by the non-short loop is shorter than 1 Mb, the loop is removed and its sequence is incorporated into the contracted node, as a unit of the model sequence.

### Scaffolding

The graph *G* = multiDBConsensus_*k*_(Reads^*^) often contains pairs of isolated edges (or pairs of tips) that together represent a contiguous region of a genome that was broken into two contigs due to a drop in coverage. Such isolated edges (as well as tips) represent *consecutive contigs* that the operation Connect(G,Reads^*^) attempts to reconnect either via small overlaps between contigs or bridging reads that bridge a gap between consecutive contigs. We thus consider the contig-set spelled by all isolated edges and tips in the graph multiDBConsensus_*k*_(Reads^*^) and analyze their prefixes and suffixes of length *L* that form the string-set *OpenEnds*_*L*_.

The operation Connect(G,Reads^*^) attempts to identify weakly-overlapping contigs in *OpenEnds*_*L*_ (for *L* = 5,000) by constructing the graph DB_*k*_(*OpenEnds*_*L*_) with a small *k*-mer size (default *k* = 500) using the jumboDBG module of LJA [22]. This graph is further simplified using the graph simplification operations of the CG algorithm. If the suffix of one contig in *OpenEnds*_*L*_ is connected to the prefix of another via an isolated edge in the resulting graph, the two contigs are merged into a longer contig. Then, MGA repeats the procedure for (*L* = 5,000,*k* = 300) and (*L* = 20,000,*k* = 500). MGA detects larger overlaps up to 20,000 bp using *k* = 500 instead of *k* = 300 to keep the graph DB_*k*_(*OpenEnds*_*L*_) relatively simple.

Next, Connect(G,Reads^*^) checks whether any two strings in *OpenEnds*_*L*_ (for *L* = 20,000) can be connected by bridging reads that bridge small gaps between the corresponding non-overlapping contigs. MGA aligns all reads to all strings in *OpenEnds*_*L*_ using minimap2 with the “map-hifi” option and analyzes all alignments with percent identity ≥ PI_span_low_ (default 90%) and a span of at least 3 kb. A read *spans* strings *S* and *T* in *OpenEnds*_*L*_ if (i) it aligns to both a prefix of *S* (starting coordinate ≤ 20) and a suffix of *T* (distance from alignment’s end to the end of string *T* ≤ 20) and (ii) at least one of these alignments has a high percent identity ≥ PI_span_high_ (default 99.5%). Bridging reads define pairs of contigs that are merged into a longer contig with the gap between them filled with the sequence of this gap in a bridging read. Allowing one end of the read to align with a lower PI_span_low_ than the other end (≥ PI_span_high_) accounts for the possibility that the two contigs belong to different haplomes of the same chromosome. In such cases, a read from one haplome will have high PI to the contig from the same haplome and lower PI to the contig from the other haplome.

### Deduplication

Because MGA is based on a phased assembly graph, sequences from the two haplomes of the same chromosome may already be separated in the initial graph and thus cannot be merged into a consensus sequence using the graph simplification approach. We classify a contig as *cognate* if it is largely contained within the longer contig, with the two sequences in such a pair usually originating from different haplomes. In this context, the shorter contig is referred to as the cognate contig of the longer *essential* contig. The function Deduplicate(*G*) identifies all cognate contigs and removes them from the assembly graph *G* using the following procedure. For all contigs spelled by edges in the *G*, the deduplication module performs all-vs-all alignments using minimap2 with options “-x asm20” and “-p 0.1”. A contig A is classified as a cognate contig of contig B if: (i) A is aligned to B with percent identity ≥ *PI*_*Cognate*_ (default: 70%); (ii) the aligned fraction on A is larger than *Span*_*Cognate*_ (default 85%); and (iii) A is shorter than B. The edge of A is then removed in *G*, and the non-branching paths in the resulting graph are condensed.

## Supplementary Information

Additional file 1: Supplementary Text and Figures. Description: Fig. S1-S22, Table S1-S7, and Supplementary Notes 1-16.

## Data Availability

Reference haplomes of the human chromosome 21 were downloaded from NCBI GenBank accessions GCA_021951015.1 [95] and GCA_021950905.1 [96]. The diploid assemblies and HiFi reads of the Pt genome were obtained from NCBI BioProject accession PRJNA725323 [97]. The diploid assemblies of the Pst genome were downloaded from NCBI GenBank GCA_039519205.1 [98] and GCA_039519225.1 [99]. HiFi reads of the Pst genome were downloaded from NCBI SRA accession SRX22053151 [100]. T2T diploid assembly of the Bonobo genome was downloaded from "Pan paniscus v2.0" in the GitHub repository https://github.com/marbl/Primates [101]. HiFi reads of Bonobo were obtained from the NCBI SRA accession SRX25393918 [102]. T2T diploid genome of HUMAN was downloaded from "hg002v1.1.fasta.gz" in the GitHub repository https://github.com/marbl/HG002 [103]. We used the HiFi sequencing data m84005_220827_014912_s1.hifi_reads.fastq.gz, m84031_231217_034919_s2.hifi_reads.fastq.gz, and m84031_231217_062403_s3.hifi_reads.fastq.gz of HUMAN from the GitHub page https://github.com/marbl/HG002/blob/main/Sequencing_data.md [103]. The T2T paternal haplome of SHEEP was downloaded from NCBI RefSeq assembly GCF_042477335.2 [104]. The T2T diploid genome and the HiFi sequencing of GIRAFFE were downloaded from GenomeArk https://www.genomeark.org/t2tall/Giraffa_tippelskirchi.html [105]. MGA is developed on the phased assembler LJA and is available at https://github.com/ZhangZhenmiao/MGA [106] under the BSD 3-Clause License. All analysis scripts were uploaded to the directory “analysis” under “src” of the MGA GitHub repository. The code used to create results in this manuscript is available at 10.5281/zenodo.20247563 [107].
