# Supplementary material for: MGA: a tool for haplotype-mixed assembly of long and accurate reads
Source: Genome Biol. 2026 Jun 17;27:201. doi: 10.1186/s13059-026-04128-5 (PMC13273997; doi:10.1186/s13059-026-04128-5)
Supplement: Supplementary file 1 — Additional file 1: Supplementary Text and Figures. Description: Fig. S1-S22, Table S1-S7, and Supplementary Notes 1-16. [file 13059_2026_4128_MOESM1_ESM.pdf]

Supplementary Text and Figures

Contents

Supplementary Figures **2**

Supplementary Tables **5**

Supplementary Notes **6**

Supplementary Note 1: Three types of genome assemblers 6

Supplementary Note 2: Information about BONOBO, HUMAN, GIRAFFE, and SHEEP datasets 6

Supplementary Note 3: Benchmarking assembly tools 7

Supplementary Note 4: Analyzing phase-switches 8

Supplementary Note 5: The VirtualHaplome approach for evaluating quality of haplotype-mixed assemblies 9

Supplementary Note 6: Evaluating MGA and hifiasm assemblies using the CRAQ tool 10

Supplementary Note 7: Evaluating MGA and hifiasm assemblies using the VirtualHaplome approach 11

Supplementary Note 8: Assembly graphs 12

Supplementary Note 9: Evaluating consensus assemblies 20

Supplementary Note 10: Exploring short contigs in MGA assemblies 21

Supplementary Note 11: Dot plots 22

Supplementary Note 12: Hifiasm assembly of bonobo chromosome 22 30

Supplementary Note 13: Consensus Genome algorithm 30

Supplementary Note 14: Inferring read coverage of edges in the multiplex de Bruijn graph 30

Supplementary Note 15: Repairing broken paths 31

Supplementary Note 16: Estimating heterozygosity rate 34

References **35**

Supplementary Figures

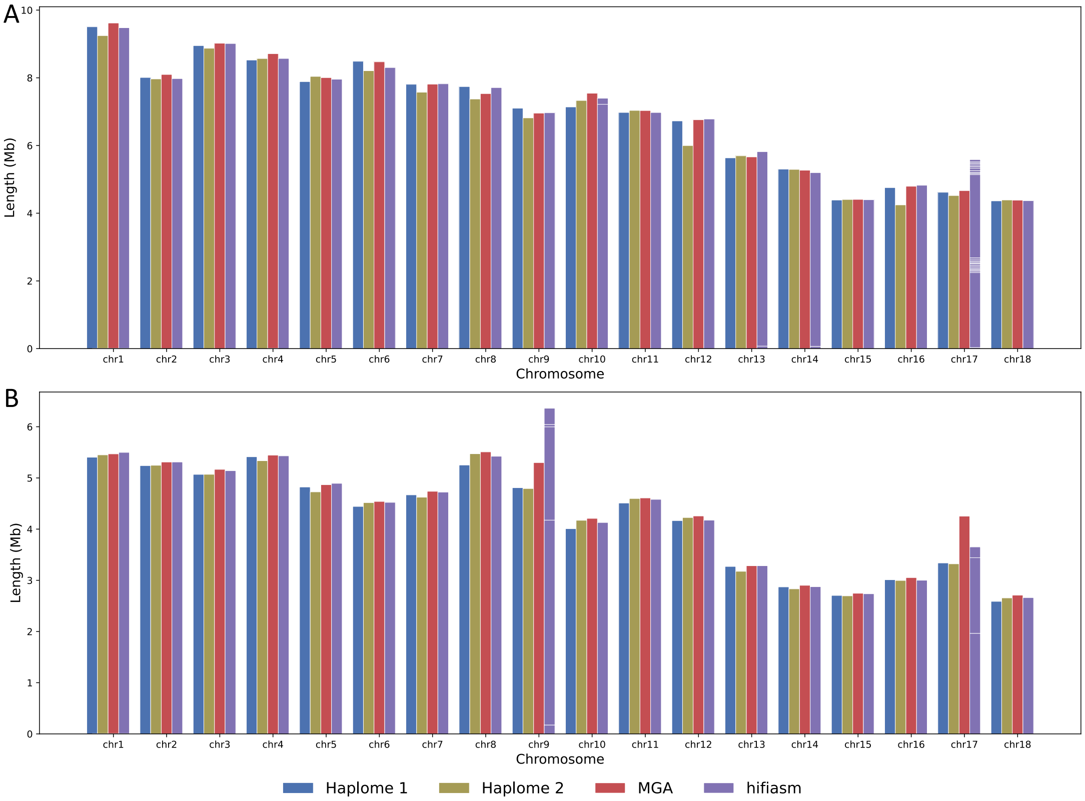

Fig. S1. The bar plot of lengths of MGA and hifiasm essential contigs for RUST (A), RUST-Pst (B) datasets. MGA generated near-complete consensus assemblies for all chromosomes of RUST and RUST-Pst. For chromosome 17 of RUST and chromosomes 9 and 17 of RUST-Pst, MGA produced consensus assemblies with higher contiguity than hifiasm. Chromosome 17 of both the *Pt* and *Pst* genomes contains rDNA tandem repeats that the published diploid assemblies failed to resolve (Genome$_{\text{RUST}}$ assembled haplome 1 of chromosome 17 into a scaffold of 12 contigs and haplome 2 into 6 contigs; Genome$_{\text{RUST-Pst}}$ assembled both haplomes of chromosome 17 into scaffolds of two contigs). For chromosome 17 of RUST-Pst, MGA produced a consensus contig substantially longer than the corresponding haplomes, suggesting that the hifiasm-based phased haplome sequence failed to assemble the large tandem repeat.

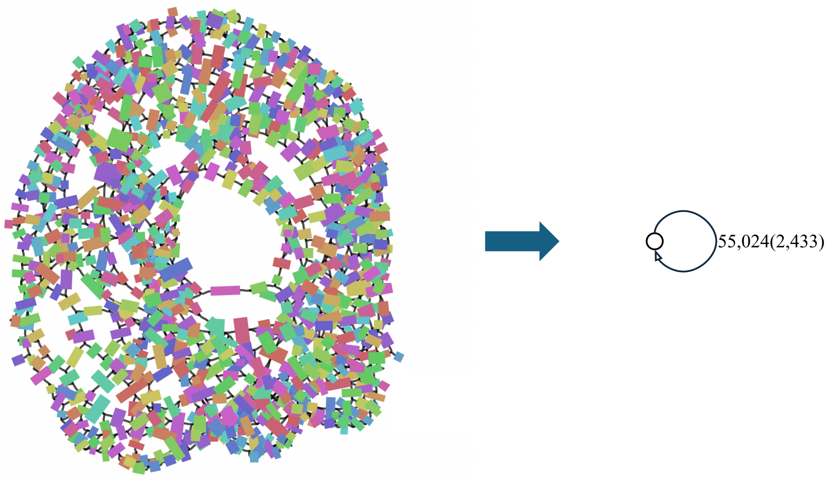

Fig. S2. A complex mtDNA subgraph in LJA_{5001}(RUST) is transformed into a loop-edge in the graph LJA_{5001}^{+}(RUST). The circular mtDNA exhibits high read coverage in the RUST dataset and forms a complex component in the graph LJA_{5001}(RUST) with 684/991 nodes/edges (visualized using the Bandage tool). The high-coverage edges (coverage ≥ 10×*Cov*) in this graph correspond to the genome path, whereas the remaining edges are artifacts resulting from a known deficiency of LJA—incomplete error correction in high-coverage regions. mtDNA in the high-coverage graph LJA_{5001}^{+}(RUST) forms a loop-edge of length 55,024 with coverage 2,433×. After error-correcting reads that are mapped to high-coverage regions and generating a clean read-seat $RUST^{*}$, LJA_{5001}($RUST^{*}$) assembles the mtDNA into a single loop.

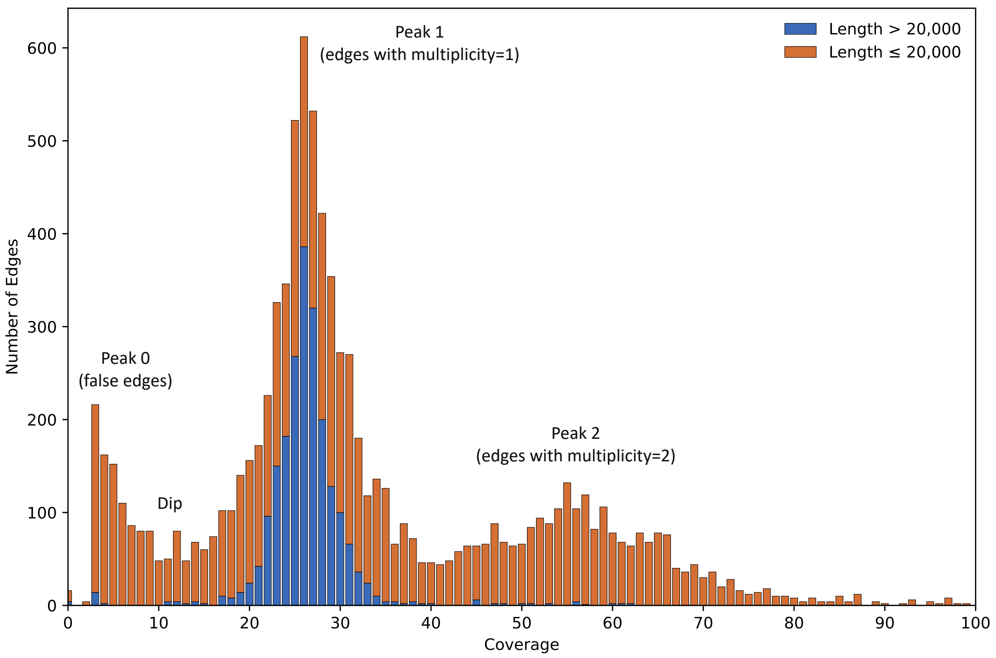

Fig. S3. Histogram of the number of edges with a given coverage in the graph LJA_{5001}(RUST). Orange (blue) bars represent the number of short (long) edges in LJA_{5001}(RUST) with a given read coverage (bin size is 1×). The histogram reveals three peaks at 4×, 27×, and 56×. The peaks centered at 27× and 56× reveal edges with multiplicities 1 and 2, respectively. The largest peak at 27× is used as an estimate of the average read coverage *Cov* of each haplome. The position of the dip between peaks at 4× and 27× is used for estimating the low coverage threshold *lowCov*. The histogram illustrates that the vast majority of edges have multiplicity 1 and the vast majority of edges of multiplicity 2 are short (and thus can potentially be resolved by constructing the multiplex graph). The vast majority of tips with multiplicity less than or equal to *lowCov* are short false edges that represent LJA artifacts.

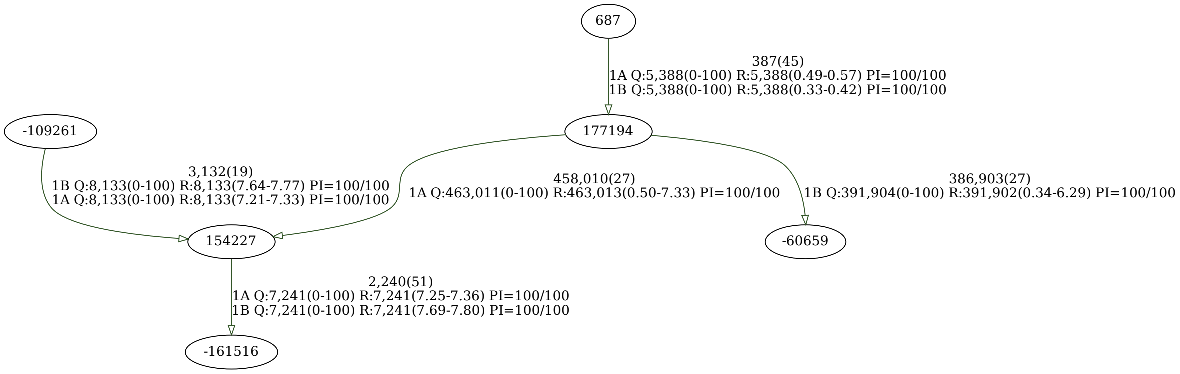

Fig. S4. A subgraph of the graph LJA_{5001}(RUST) with broken tips. An edge (177194, 154227) is preserved in one haplome but broken into an out-tip (177194, -60659) and in-tip (-109261, 154227) in another haplome. For simplicity, the Figure does not show incoming edges into node 687 and outgoing edges from node -161516.

Supplementary Tables

Table S1. The statistics of MGA graph transformations.

Graph	RUST (#nodes/edges/long edges)	RUST-Pst (#nodes/edges/long edges)	BONOBO (#nodes/edges/long edges)	HUMAN (#nodes/edges/long edges)	SHEEP (#nodes/edges/long edges)	GIRAFFE (#nodes/edges/long edges)
**LJA$_k$(*Reads*)**	7530/ 10967/ 2145	9028/ 12852/ 2520	150750/ 225320/ 70410	240594/ 360404/ 71926	14640/ 21384/ 4488	194026/ 290648/ 69248
**DB$_k$(*Reads**)**	5884/ 8593/ 2081	6886/ 10152/ 2520	149522/ 223596/ 70542	238620/ 357518/ 72076	12462/ 18314/ 3888	194148/ 290838/ 69234
**DB$_k$(*Genome*)**	5396/ 7949/ 1983	6142/ 9078/ 2512	132096/ 197984/ 62600	237652/ 356304/ 69356	NA	189342/ 283958/ 67772
**multiDBG$_k$(*Reads**)**	1411/ 2014/ 918	1658/ 2622/ 1772	9160/ 15382/ 13890	31276/ 56652/ 54236	3390/ 4974/ 2678	17112/ 28666/ 27382
**MGA$_k$(*Reads**)**	126/ 78/ 52	92/ 56/ 38	294/ 160/ 142	154/ 92/ 88	262/ 156/ 132	142/ 76/ 68

All values are computed for k=5001. The DB$_k$(*Genome*) row (generated by jumboDBG) was added to illustrate that graphs DB$_k$(*Genome*) and DB$_k$(*Reads**) have similar sizes. All statistics given in this Table count both the direct and the reverse strands.

Table S2. The running time and memory footprint for MGA, Falcon, Flye, HiCanu, hifiasm, and LJA+purge_dups on the RUST dataset.

Assembler	MGA	Falcon	Flye	HiCanu	hifiasm	LJA + purge_dup
**System time (seconds)**	3248	185075	12326	47281	826	2075 + 152
**User time (seconds)**	5034126	416849	406260	1371066	263464	4896970 + 59965
**Elapsed time (h:mm:ss)**	35:37:30	5:30:34	7:24:41	8:31:03	1:42:15	34:15:44 + 1:24:10
**Memory (Mb)**	55536	25074	47298	16870	19704	55536

All assemblers were run using default parameters for assembling HiFi reads if not specified and the thread numbers were set to 50 if possible (for MGA, Flye, and hifiasm). Hifiasm was run with option "--primary". MGA launches LJA twice, the first time to construct the graph LJA$_k$(*Reads*) and the second time to construct the graph multiDB$_k$(*Reads**). However, the running time of MGA is dominated by the first run when LJA constructs the graph LJA$_k$(*Reads*) because the running time of LJA is dominated by the error-correction step. The second launch of LJA is fast because it constructs the MDB graph from the already error-corrected clean read-set *Reads**. A large MGA runtime on the RUST dataset is likely caused by extensive error corrections in this highly-polymorphic dataset that slowed down LJA.

Supplementary Notes

Supplementary Note 1: Three types of genome assemblers

Fig. S5 illustrates the concepts of phased, haplotype-resolved, and haplotype-mixed assemblies. The de Bruijn graph-based assemblers—such as Verkko and LJA—first construct a phased multiplex de Bruijn graph. Verkko2 [1] transforms the phased DB graph into a diploid DB graph (using complementary sequencing technologies such as Hi-C reads) while MGA transforms it into a haplotype-mixed DB graph. Flye uses a different concept of the A-Bruijn graph [2] to construct an analog of the phased de Bruijn graph that collapses highly similar regions of two haplomes. Thus, Flye is a phased assembler with some features of a haplotype-mixed assembler. Canu, Falcon, and hifiasm are string-graph based assemblers that construct a phased assembly (hifiasm further transforms it into a consensus assembly).

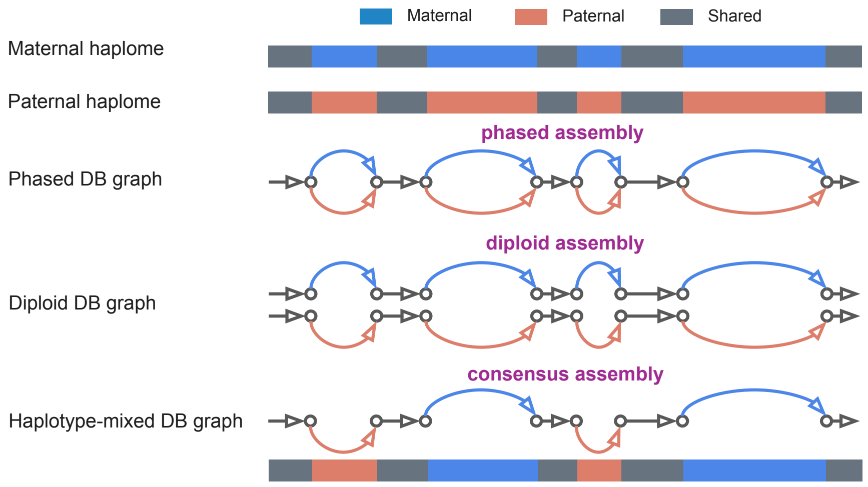

Fig. S5. Illustration of the concepts of phased, haplotype-resolved (diploid), and haplotype-mixed (consensus) assemblies.

Supplementary Note 2: Information about BONOBO, HUMAN, GIRAFFE, and SHEEP datasets

Below we provide information about the HUMAN, BONOBO, GIRAFFE, and SHEEP datasets.

BONOBO dataset. We downloaded the HiFi reads and the T2T bonobo genome from the sources specified in [3]. The reference genome was assembled by Verkko using HiFi reads, ultralong ONT reads, Hi-C reads, and parent-child trio Illumina reads.

HUMAN dataset. This dataset was downloaded from https://github.com/marbl/HG002/blob/main/Sequencing_data.md, including m84005_220827_014912_s1.hifi_reads.fastq.gz, m84031_231217_034919_s2.hifi_reads.fastq.gz, and m84031_231217_062403_s3.hifi_reads.fastq.gz. We used “hg002v1.1.fasta.gz” downloaded from https://github.com/marbl/HG002 as the reference T2T genome. The reference genome was assembled by Verkko using HiFi reads and ultra-long ONT reads.

GIRAFFE dataset. We downloaded the HiFi reads and the T2T giraffe genome from the GenomeArk (https://www.genomeark.org/t2t-all/Giraffa_tippelskirchi.html). Kim et al., 2025 [4] assembled the reference genome with Verkko using HiFi reads, ONT ultra-long reads, ONT duplex data, and Hi-C data.

SHEEP dataset. The paternal sheep haplome was downloaded from NCBI RefSeq assembly GCF_042477335.2. Olagunju et al., [5] assembled this haplome by Verkko using HiFi reads, ONT reads, ONT ultra-long reads, and Hi-C reads. The maternal haplome has not yet been assembled and released at the time of manuscript preparation.

The estimated heterozygous mutation rate for these genomes varies from 0.12% to 0.14% (Supplementary Note 16).

Supplementary Note 3: Benchmarking assembly tools

Information about assembly tools. Assembly tools HiCanu (v2.2), hifiasm (git commit fc98214), Flye (v2.9.5), Falcon (v1.8.1), and LJA+purge_dup (v1.2.6) were used for benchmarking. All assemblers were run with their default parameters for HiFi datasets if not specified. Hifiasm was run with the option "--primary" for generating primary assembly. Falcon was run based on https://github.com/PacificBiosciences/pb-assembly/blob/master/cfgs/fc_run_HiFi.cfg [6]. To ensure compatibility with Falcon's fasta2DB, RUST reads were renamed to the format "[name]/[number]/0_[readlength]" for running Falcon. We ran LJA using the git commit fb31d15 from its experimental branch.

Assembling genomes with reduced coverage. We simulated three HiFi datasets ($RUST_{sim}$) with 29x, 25x, and 20x coverages from the two haplomes of the largest chromosome in $Genome_{RUST}$ (9.2 Mb and 9.5 Mb). Table S3 shows assembly statistics of MGA and hifiasm on the HiFi datasets simulated using pbsim3 (v3.0.5). Both MGA and hifiasm generated accurate and contiguous haplotype-mixed assemblies for datasets with 29x and 25x coverages. MGA obtained better assembly contiguity than hifiasm on $RUST_{sim}$ with 20x coverage. For all three datasets, MGA achieved lower phase-switch rates than hifiasm.

Table S3. Comparing hifiasm and MGA assemblies with reduced read coverage ($RUST_{sim}$ dataset).

Assembler	#Contigs (essential contigs only)	#Long contigs (≥ 1Mb)	Total contig length (Mb)	N50 (Mb)	N90 (Mb)	Phase-switch rate
**$RUST_{sim}$** (29x, 1 chromosome, haplome sizes 9.2 Mb and 9.5 Mb)						
**MGA**	1	1	9.4	**9.4**	**9.4**	**1.2%**
**hifiasm**	1	1	9.3	9.3	9.3	2.1%
**$RUST_{sim}$** –25 (25x, 1 chromosome, haplome sizes 9.2 Mb and 9.5 Mb)						
**MGA**	1	1	9.6	**9.6**	**9.6**	**1.8%**
**hifiasm**	1	1	9.5	9.5	9.5	2.4%
**$RUST_{sim}$**–20 (20x, 1 chromosome, haplome sizes 9.2 Mb and 9.5 Mb)						
**MGA**	1	1	9.2	**9.2**	**9.2**	**0.5%**
**hifiasm**	2	2	9.3	5.7	3.6	2.7%

Supplementary Note 4: Analyzing phase-switches

Given a read-set *Reads* derived from a genome formed by haplomes $Haplome_1$ and $Haplome_2$ and a consensus assembly (virtual haplome) *A*, our goal is to identify *phase-switches* between $Haplome_1$ and $Haplome_2$ within the virtual haplome *A*. The number of phase switches can be estimated indirectly using the yak tool [7] by generating *k*-mer spectra of $Haplome_1$ and $Haplome_2$ and performing the *k*-mer-based analysis commonly usually trio binning [8]. Instead, we directly estimate the number of phase-switches in the consensus assembly using GraphAligner v1.0.19 [9].

We first construct the graph $DB_k(Haplome_1+Haplome_2)$ using jumboDBG module of LJA and label each edge (v,w) in this graph by the labels of chromosomes in $Haplome_1$ and $Haplome_2$ where seq(v,w) occurs (e.g., $Genome_{RUST}$ has 18×2 labels for 18 pairs of chromosomes). Afterwards, we align each chromosome in the consensus assembly against this graph using GraphAligner [9]. We note that GraphAligner may split an alignment of a single consensus chromosome into a series of local sub-alignments and that some of the resulting sub-alignments may have a rather low percent identity. We thus retain sub-alignments with percent identity at least 90%.

Since each chromosome in the consensus assembly represents a path in the graph $DB_k(Haplome_1+Haplome_2)$, we compute the number of phase-switches by analyzing labels of edges in this path. For example, consider a path representing chromosome 7 (haplomes 7A and 7B) in the consensus assembly with edges labeled as follows:

7A→7A→7A/7B→7A→7A/7B **➔**7B→7A/7B→7B→7A/7B**➔**7A→7A/7B

This path has 2 phase-switches shown by bolded arrows. A more complex situation arises when such a path contains edges that are labeled by multiple chromosomes such as an edge 3A/B below that adds two *erroneous switches*:

7A**➔**3A/3B**➔**7A→7A/7B→7A→7A/7B/5A **➔**7B→7A/7B→7B/9A→7A/7B**➔**7A→7A/7B

The *phase-switch rate* is defined as the ratio of the number of phase-switches to the total number of edge-transitions in the paths representing each chromosome in the consensus assembly.

Chromosome 17 of the *Pt* genome contains extra-long rDNA repeat [10] that the diploid assembly $Genome_{RUST}$ failed to resolve (11 and 5 gaps in haplome 1 and haplome 2, respectively). Most consensus assemblers also failed to resolve it, e.g., hifiasm assembled this chromosome into two long contigs (defined as contigs longer than or equal to 1 Mb) and a few short contigs (Fig. S1). Consequently, the GraphAligner resulted in a very large number of edge transitions in chromosome 17 that greatly affected the estimate of the phase-switch rate. Therefore, chromosome 17 of $Genome_{RUST}$ was excluded from the phase-switch rate calculation.

Since GraphAligner crashed on the large BONOBO, HUMAN, GIRAFFE and SHEEP datasets, the phase-switch rates for these datasets were evaluated using a less accurate yak tool.

Supplementary Note 5: The VirtualHaplome approach for evaluating quality of haplotype-mixed assemblies

Given a parental and a maternal haplome H_1 and H_2, we can generate their *virtual haplome* by “recombining” them, i.e., by generating an arbitrary traversal of the phased de Bruijn graph $DB_k(H_1+H_2)$. Since many such virtual haplomes are possible (Fig. S6 presents four virtual haplomes), our goal is to identify an *A-virtual haplome* for a haplotype-mixed assembly A: a virtual haplome that is most similar to A.

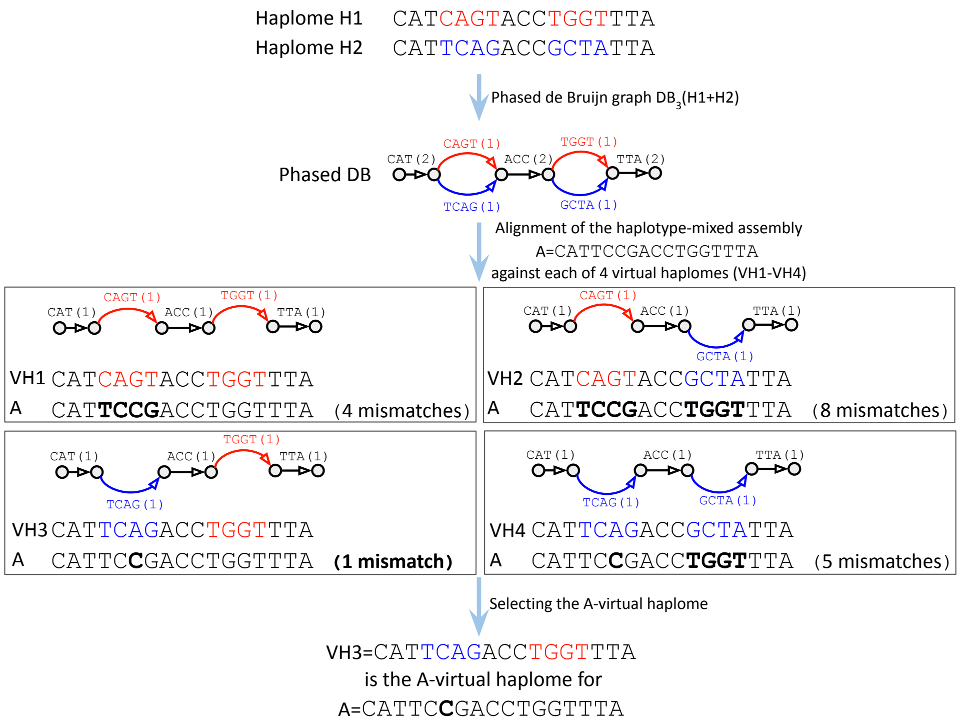

Fig. S6. Identifying an *A*-virtual haplome for a haplotype-mixed assembly *A*. In the phased de Bruijn graph, the sequences of black edges include both starting and ending nodes. The sequences of red and blue edges exclude starting and ending nodes.

The QUAST-LG tool [11] compares a newly generated haplome assembly against the reference haplome and thus allows one to compare the quality of various assemblers. However, since there is no reference haplome in the case of a haplotype-mixed assembly A, its quality should be compared against an A-virtual haplome. Below we describe how to generate this virtual haplome and thus apply QUAST-LG for assessing the quality of a haplotype-mixed assembly.

Given a haplotype-mixed assembly A of haplomes H_1 and H_2, we define an *A-virtual haplome* as a virtual haplome of H_1 and H_2 that is most similar to A. Below we describe how to construct an A-virtual haplome by aligning assembly A to the graph $DB_k(H_1+H_2)$ using GraphAligner [9] and illustrate this construction using haplomes of chromosome 21 in the bonobo genome.

Since GraphAligner becomes slow and less accurate in the case of large graphs, we selected a large k-mer size (k=20,001) and constructed a *sparse de Bruijn graph* SDB(H_1+H_2, *Anchors*)

where the anchor-set *Anchors* consists of all 20,001-mers that occur only once in H_1 and only once in H_2 [12]. This graph (that is smaller than $DB_k(H_1+H_2)$) contains only 140/202 nodes/edges. Since the sparse de Bruijn graph may have pseudo-bubbles with identical legs, we further collapsed such pseudo-bubbles.

Below, we refer to the sequence of chromosome 21 in the MGA and hifiasm haplotype-mixed assemblies of the BONOBO dataset as MGA(BONOBO21) and hifiasm(BONOBO21), respectively.

Ideally, GraphAligner should generate a single path representing an alignment of MGA(BONOBO21) and hifiasm(BONOBO21) against the graph SDB(H_1+H_2, *Anchors*). However, even with this relatively small graph, GraphAligner generated multiple alignment paths through SDB(H_1+H_2, *Anchors*) for both MGA(BONOBO21) and hifiasm(BONOBO21). Some of these paths overlap and some are contained within each other—after merging overlapping paths and removing paths contained within other paths, we generated 8 paths that cover 88.9% of MGA(BONOBO21) and 4 paths that cover 75.6% of hifiasm(BONOBO21).

We used the "corrected-out" parameter of GraphAligner that replaces the sequences in the haplotype-mixed assembly *A* with the sequences of their corresponding paths in the graph and fills the segments between consecutive paths with the segments in the MGA and hifiasm assemblies, respectively. We refer to the resulting "corrected consensus assembly" as a single *A*-virtual haplome and use it as the reference for QUAST-LG to assess the quality of the haplotype-mixed assembly *A*.

Supplementary Note 7 compares the quality of MGA and hifiasm assemblies using the VirtualHaplome approach.

Supplementary Note 6: Evaluating MGA and hifiasm assemblies using the CRAQ tool

CRAQ [13] is a reference-free tool for evaluating the assembly quality of haplotype-mixed assemblies. It aligns reads to the assembly, classifies assembly errors as *regional errors* (CRE) or as *structural errors* (CSE), reports assembly quality metrics (herein referred to as AQI_CRQ and AQI_CSE) for these two types of errors, and further outputs the overall AQI metric for an assembly (herein referred to as AQI_Overall). The heterozygous sites between haplotypes are not classified as assembly errors because CRAQ differentiates assembly errors from heterozygosity. CRAQ classifies assemblies with AQIs over 90 as *reference quality* assemblies, and 80-90 as *high quality* assemblies (https://github.com/JiaoLaboratory/CRAQ).

Our analysis revealed that CRAQ suffers from misclassifications of phase switches as assembly errors. To illustrate this point, we used CRAQ to analyze the maternal haplome of the BONOBO dataset assembled by [3]. Since this T2T haplome (viewed as a "perfect" haplotype-resolved assembly) has no phase switches or assembly errors, CRAQ is expected to output AQIs equal to 100. However, it assessed the quality of this assembly as AQI_CRE=99.8, AQI_CSE=88.5, and AQI_Overall=93.8.

These statistics indicate that CRAQ reports many false positive assembly errors—and, to this end, that assemblies with an AQI_CSE above 80 should be viewed as very accurate. Also, incomplete or highly fragmented assemblies may be evaluated as highly accurate since they provide fewer opportunities to detect misassemblies. This observation may explain why AQIs for more fragmented and less complete hifiasm assemblies are slightly higher than for MGA assemblies.

We used the CRAQ (v1.10) tool to evaluate the assembly quality of the MGA and hifiasm assemblies of the fungi and mammalian datasets (Table S4). MGA and hifiasm obtained comparable AQIs for all datasets, although hifiasm's AQIs tended to be slightly higher than MGA's. Possible reasons for these differences include MGA's assemblies being less fragmented and MGA's use of repeat models to represent long HRRs.

Table S4. Evaluation of MGA and hifiasn assemblies using the CRAQ tool.

Assembler	AQI_CRE	AQI_CSE	AQI_Overall
		**RUST**	
**MGA**	96.730	85.074	89.547
**hifiasm**	96.745	88.599	92.493
		**RUST-Pst**	
**MGA**	95.388	80.499	87.313
**hifiasm**	94.796	82.434	88.184
		**HUMAN**	
**MGA**	97.517	89.599	93.390
**hifiasm**	99.448	91.966	95.561
		**BONOBO**	
**MGA**	98.351	85.074	91.232
**hifiasm**	99.358	86.968	92.751
		**SHEEP**	
**MGA**	99.574	88.409	93.660
**hifiasm**	99.526	91.110	95.132
		**GIRAFFE**	
**MGA**	99.371	90.899	94.946
**hifiasm**	99.648	92.830	96.118

AQI_CRQ, AQI_CSE, and AQI_Overall statistics measure the assembly quality with respect to clipping-based regional errors, clipping-based structural errors, and clipping-based overall quality, respectively. We used the default CRAQ parameters and applied CRAQ to essential contigs only.

Supplementary Note 7: Evaluating MGA and hifiasm assemblies using the VirtualHaplome approach

For an objective comparison, we applied the VirtualHaplome approach only to bonobo chromosomes that were assembled into near-complete contigs by both MGA and hifiasm (chromosomes 9, 11, and 21). MGA demonstrated higher structural accuracy, with only 1 total misassembly compared to 11 for hifiasm, yielding higher NA50/NGA50 metrics (Table S5). MGA maintained lower misassembly counts despite having a higher percentage of its assembly subjected to analysis (93.6% versus 78.8% on average). Because 6.4% (MGA) and 21.2% (hifiasm) of the virtual haplomes were identical to the consensus—and therefore incapable of

revealing error—the evaluation is biased in favor of hifiasm, likely explaining its marginally better base-pair accuracy (for chromosomes 11 and 21) and local alignment accuracy (for chromosomes 9 and 11) on a subset of chromosomes.

Table S5. The assembly statistics for bonobo chromosomes 9, 11 and 21 that were assembled into a single near-complete contig by both MGA and hifiasm.

**Chromosome**	**Chromosome 9**		**Chromosome 11**		**Chromosome 21**	
**Assembler**	MGA	hifiasm	MGA	hifiasm	MGA	hifiasm
**Total length (Mb)**	136.7	135.9	125.7	127.6	78.3	77.2
**Coverage by GraphAligner paths**	**95.8%**	87.7%	**96.2%**	73.1%	**88.9%**	75.6%
**NA50 (Mb)**	**131.5**	103.0	**125.7**	81.8	**78.3**	26.5
**# misassemblies (relocations + translocations + invertions)**	**1 (1+0+0)**	2 (2+0+0)	**0 (0+0+0)**	4 (4+0+0)	**0 (0+0+0)**	5 (5+0+0)
**# local misassemblies**	3	**0**	2	**0**	**32**	38
**# mismatches per 100 kbp**	**1.90**	2.35	4.39	**3.67**	8.95	**3.91**
**# indels per 100 kbp**	2.58	**1.29**	3.26	**1.59**	3.99	**1.16**

NGA50=NA50 for all assemblies.

Supplementary Note 8: Assembly graphs

- Fig. S7 presents the graph DB_{5001}(H21P+H21M) after iteratively processing all simple bubbles and whirls.
- Fig. S8 presents the component of chromosome 10 of the contracted bubble-collapsed graph LJA_{5001}(RUST).
- Fig. S9 presents the largest weakly-connected components of the contracted bubble-collapsed graph LJA_{5001}(RUST) and LJA_{5001}(RUST-Pst).
- Fig. S10 presents near-complete consensus chromosomes assembled by MGA for RUST and RUST-Pst assemblies.
- Fig. S11 presents near-complete consensus chromosomes assembled by MGA for BONOBO, HUMAN, GIRAFFE, and SHEEP assemblies (suffixes M/P denote the maternal/paternal haplomes).
- Fig. S12 presents a small connected component in the graph LJA_{5001}(BONOBO) after iterative simple bubble collapsing.
- Fig. S13 presents the subgraph representing the rDNA repeat before (top) and after (bottom) contracting short edges in chromosome 17 of RUST-Pst.

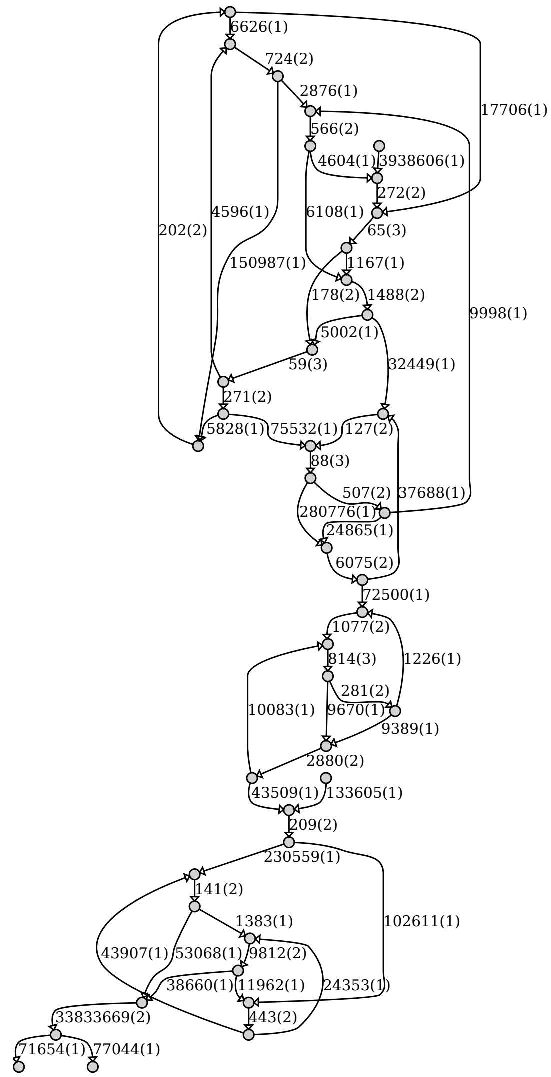

Fig. S7. The graph DB_{5001}(H21P+H21M) after iteratively processing all simple bubbles and whirls. The Figure shows the subgraph with 40/56 nodes/edges representing one of two strands (omitting the reverse complements). Each edge is labeled with its length and multiplicity.

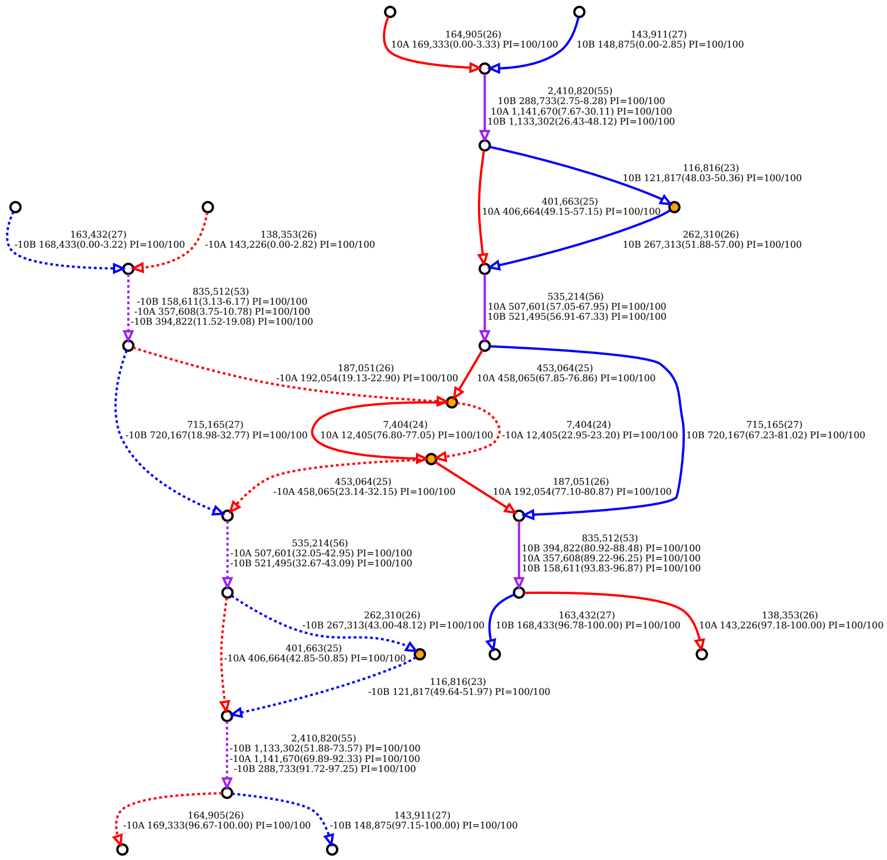

Fig. S8. A component of the contracted bubble-collapsed graph LJA$_{5001}$(RUST) representing haplomes of chr10 of the *Pt* genome. This component is formed by reads from the chromosomes 10A and 10B, as well as their reverse strands, -10A and -10B. Contracted nodes are colored orange. Edges are coded as solid red (10A), solid blue (10B), solid purple (10A and 10B), dotted red (-10A), dotted blue (-10B), and dotted purple (-10A and -10B). Information about alignments' spans on edges is omitted for brevity.

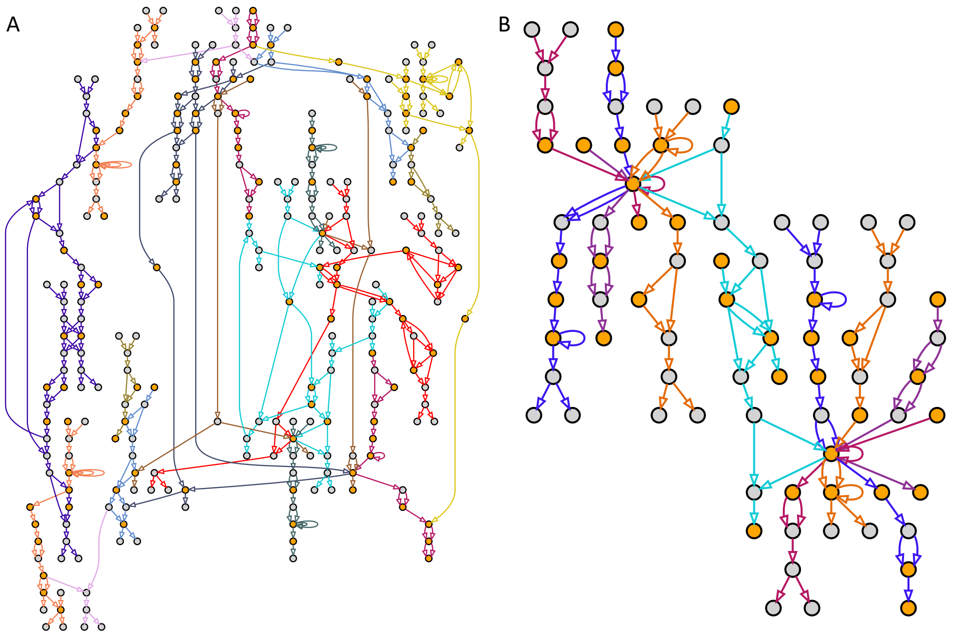

Fig. S9. The largest weakly-connected components of the contracted bubble-collapsed graph LJA$_{5001}$(RUST) (A) and LJA$_{5001}$(RUST-Pst) (B). The self-loops on contracted nodes are not removed and simple bubbles that start and end at contracted nodes remain uncollapsed. (A) The largest weakly-connected component in LJA$_{5001}$(RUST) consists of 272/364 nodes/edges, including 55 contracted nodes and their reverse complements (shown in orange). Edges are colored in twelve distinct colors corresponding to chromosomes 1, 2, 3, 4, 7, 8, 9, 11, 12, 15, 16, and 18 and edge labels are omitted for clarity. The contracted nodes in the contracted bubble-collapsed graph represent HRRs in the *Pt* genome; the largest one resulted from merging 991 short edges of total length 1,694,295 bp. (B) The largest weakly-connected component in LJA$_{5001}$(RUST-Pst) consists of 78/102 nodes/edges, including 18 contracted nodes and their reverse complements (shown in orange). Edges are colored in five distinct colors corresponding to chromosomes 4, 5, 6, 16, and 17 and edge labels are omitted for clarity.

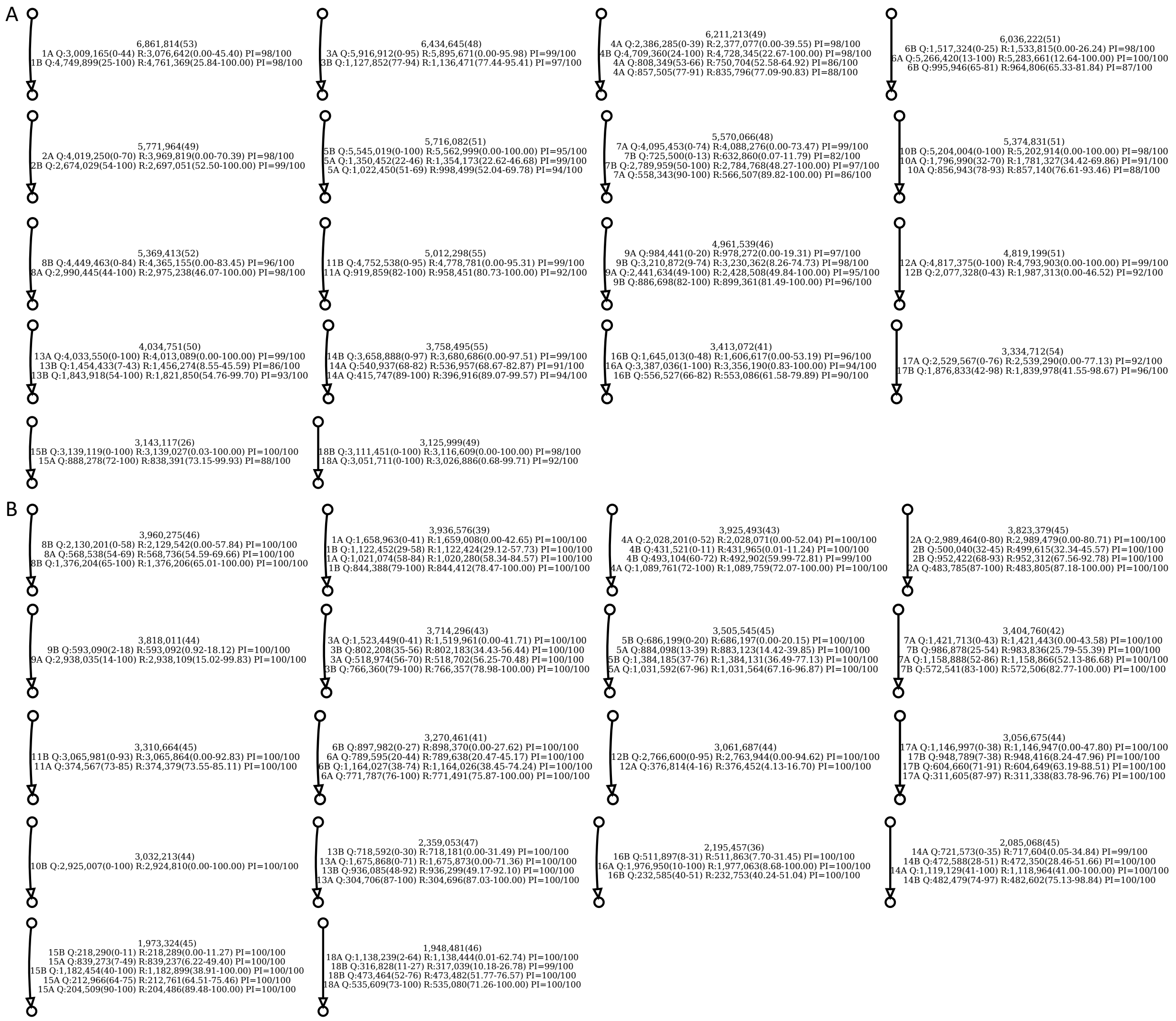

Fig. S10. **Near-complete consensus chromosomes assembled by MGA in RUST (A) and RUST-Pst (B)**. The final MGA graphs of RUST and RUST-Pst contain 18 (A) and 18 (B) long contigs representing near-complete consensus chromosomes, respectively (these contigs are ordered in descending order of length). Chromosome colors are removed for simplicity. Suffixes A/B in chromosome IDs denote haplome 1 and haplome 2. Some relatively short and highly repetitive prefixes/suffixes of consensus chromosomes do not align to the phased assemblies by minimap2. This is likely due to a known limitation of minimap2 in aligning highly repetitive regions. For instance, with "asm20" (and "-p 0.1" to allow more secondary alignments), minimap2 failed to align the end of the consensus contig of RUST chromosome 3 to the haplomes, although the dot plot (Supplementary Note 11) indicates a clear alignment.

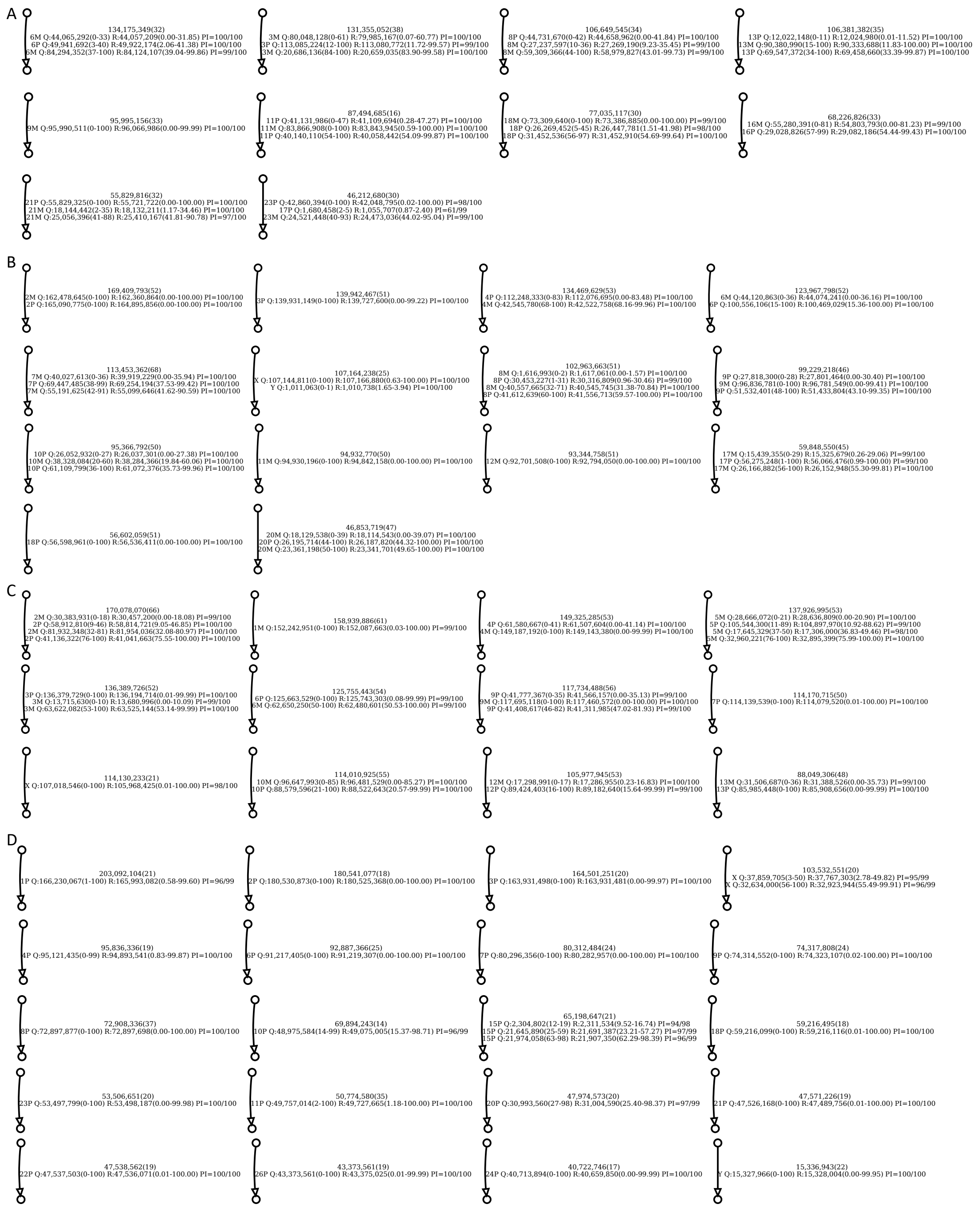

Fig. S11. Near-Complete consensus chromosomes assembled by MGA in BONOBO (A), HUMAN (B), GIRAFFE (C), and SHEEP (D) datasets. The final MGA graph of BONOBO, HUMAN, GIRAFFE and SHEEP contains 10, 14, 12, and 20 long edges representing near-complete consensus chromosomes, respectively (ordered in descending order of length; read coverages are shown in parentheses). Chromosome colors are removed for simplicity. Suffixes M/P in chromosome IDs denote maternal haplome and paternal haplome. We only aligned the consensus chromosomes to the paternal sheep haplome for SHEEP because the maternal haplome is not yet available. Some relatively short and highly-repetitive prefixes/suffixes of consensus chromosomes do not align to the reference haplomes by minimap2. This is likely due to a known limitation of minimap2 in aligning highly-repetitive regions.

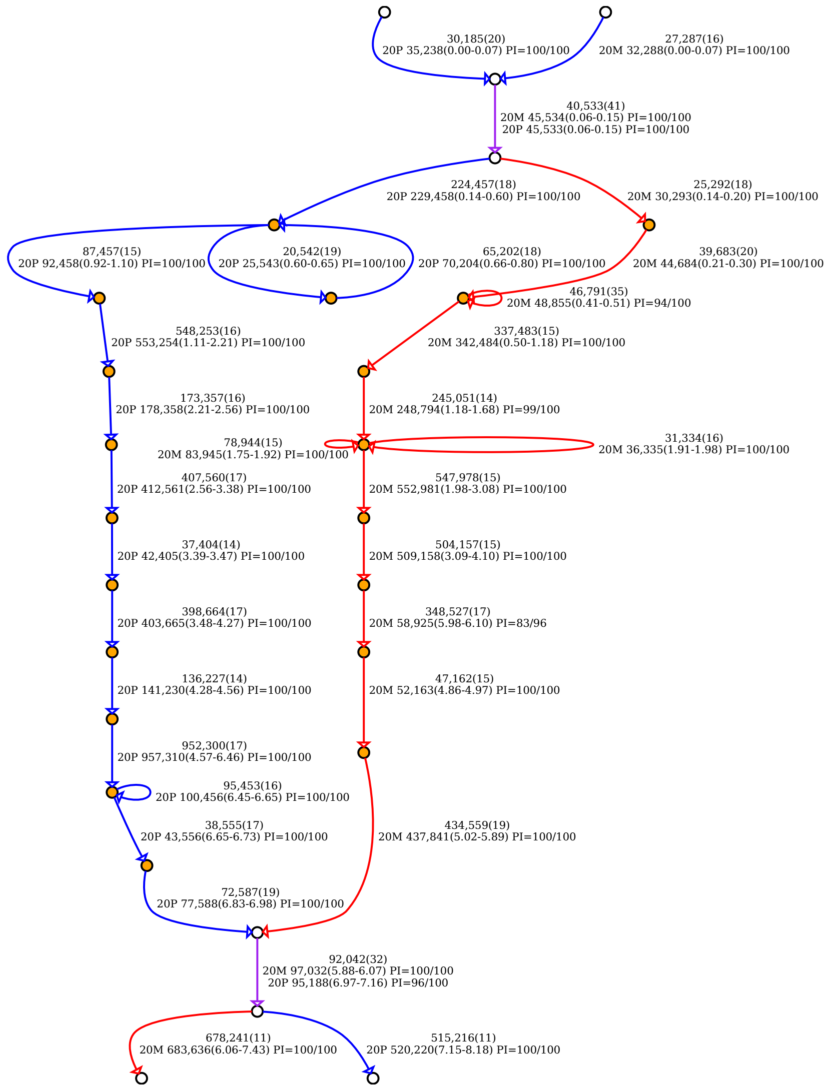

Fig. S12. A small connected component in the graph LJA$_{5001}$(BONOBO) after iterative simple bubble collapsing. Contracted nodes are shown in orange, and self-loops on contracted nodes are not removed. The suffixes M/P in the chromosome IDs denote maternal and paternal haplomes, respectively. The edges are colored into red (20M), blue (20P), and purple (20M and 20P).

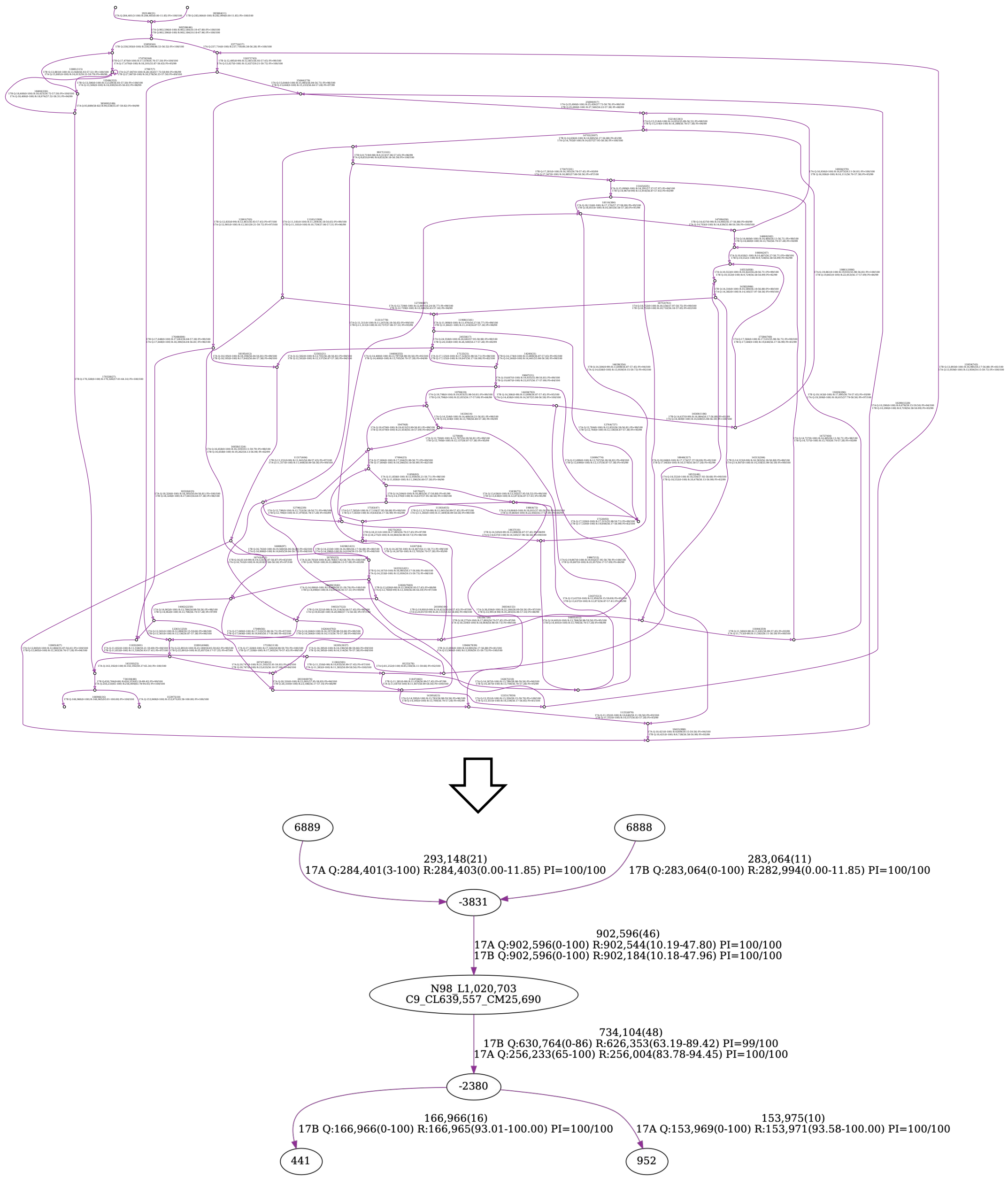

Fig. S13. The subgraph representing the long rDNA repeat in chromosome 17 of RUST-Pst before (top) and after (bottom) contracting short edges. The edge labels are annotated similarly to Figure 1. Before contracting short edges, the subgraph of the multiplex de Bruijn graph representing this chromosome has 74/113 nodes. After contracting, the rDNA repeat is represented by a single contracted node. Since this repeat is difficult (likely impossible) to resolve using HiFi reads, MGA represents it as a repeat model. The largest node "N98_L1,020,703" is a contracted node formed by contracting 98 short edges with a total length of 1,020,703 bp. These short edges do not contribute to the sequence of this node and are removed from the assembly. The contracting operation added 9 self-loops representing long edges on the contracted node, denoted "C9". The long edges have a total length of 639,557 bp, and a median length of 25,690 bp. These long edges, connected using an arbitrary order, are added to the node as a model sequence.

Supplementary Note 9: Evaluating consensus assemblies

Until recently, evaluating the quality of consensus assemblies was challenging because accurate diploid assemblies of both haplomes—needed as a reference—were unavailable. In the absence of a rigorous objective function for evaluating consensus assemblies and a QUAST-like tool for this purpose, previous quality assessment efforts were limited to metrics such as N50, N90, and BUSCO [14,15].

The availability of completely assembled haplomes ($Haplome_1$ and $Haplome_2$) enables aligning consensus assembly against them and using the resulting percent identity (PI) for each consensus contig as a quality assessment metric. However, aligning long and structurally variable sequences across their entire length using commonly used aligners such as minimap2 [16] faces challenges that are further amplified in highly-repetitive regions [17]. We thus used the UniAligner tool [17] to analyze the repetitive and highly diverged haplomes.

Table S6 summarizes the PI-based evaluation (each PI is reported in PI_1/PI_2, representing the standard PI and the PI ignoring gaps ≥10 bp) of consensus assemblies for the RUST and RUST-Pst datasets generated by MGA and hifiasm. We define the consensus PI for a chromosome in the consensus assembly as the larger of $PI_1(Consensus,Haplome_1)$ and $PI_1(Consensus,Haplome_2)$. For nearly all chromosomes, consensus PI is higher than $PI_1(Haplome_1,Haplome_2)$ for the consensus assemblies generated by either MGA or hifiasm. The average PI over $PI(Consensus,Haplome_1)$ and $PI(Consensus,Haplome_2)$ of all chromosomes for MGA (RUST=88/100, RUST-Pst=92/100) is slightly higher than that for hifiasm (RUST=86/100, RUST-Pst=91/100) and higher than that between two haplomes (RUST=78/99, RUST-Pst=88/99).

Table S6. Analysis of the RUST and RUST-Pst assemblies for mutation detection.

**Chr#**	**1**	**2**	**3**	**4**	**5**	**6**	**7**	**8**	**9**	**10**	**11**	**12**	**13**	**14**	**15**	**16**	**17**	**18**	**Avg**
**RUST**																			
**PI (Hap_1,Hap_2)**	80/99	81/99	80/99	79/99	80/99	68/98	83/100	73/99	68/98	83/99	74/99	70/99	82/100	69/99	81/99	76/100	80/99	89/100	78/99
**PI (MGA, Hap_1)**	86/100	90/100	96/100	86/100	84/99	**95/100**	**96/100**	81/99	77/99	82/99	75/99	**98/100**	**99/100**	70/99	81/99	**93/100**	**88/100**	91/100	87/100
**PI (MGA, Hap_2)**	89/100	90/100	81/99	92/100	94/100	72/99	87/100	90/100	88/100	**96/100**	98/100	71/99	82/100	**99/100**	100/100	81/100	87/100	**98/100**	89/100
**PI (hifiasm, Hap_1)**	**91/100**	**98/100**	96/100	**96/100**	84/99	77/99	95/100	**96/100**	77/99	92/100	**99/100**	88/100	93/100	94/100	100/100	89/100	45/99	93/100	89/100
**PI (hifiasm, Hap_2)**	88/100	82/99	81/99	82/100	**95/100**	89/100	87/100	75/99	88/100	90/100	75/99	79/99	90/100	73/99	81/99	84/100	52/100	96/100	83/100
**RUST-Pst**																			
**PI (Hap_1,Hap_2)**	90/100	90/100	87/99	91/100	88/99	89/99	91/100	89/99	64/99	86/99	87/99	92/100	90/99	93/100	91/99	94/100	91/100	85/99	88/99
**PI (MGA, Hap_1)**	96/100	98/100	91/100	**97/100**	94/100	94/100	**97/100**	89/100	**89/100**	87/100	88/99	93/100	97/100	97/100	94/100	98/100	66/100	93/100	92/100
**PI (MGA, Hap_2)**	95/100	92/100	**95/100**	94/100	94/100	94/100	94/100	**99/100**	60/99	**99/100**	**100/100**	99/100	93/100	95/100	96/100	95/100	**68/100**	92/100	92/100
**PI (hifiasm, Hap_1)**	96/100	98/100	94/100	95/100	**97/100**	**95/100**	96/100	92/100	52/99	93/100	92/100	99/100	97/100	95/100	95/100	95/100	57/100	**95/100**	91/100
**PI (hifiasm, Hap_2)**	94/100	92/100	92/100	96/100	91/100	93/100	94/100	97/100	82/100	93/100	95/100	93/100	93/100	**98/100**	96/100	**100/100**	56/99	90/99	91/100

The table analyzes the RUST and RUST-Pst assemblies for identifying mutations via read mapping against the reference genome. It shows percent identities $PI(Haplome_1,Haplome_2)$,

PI(Consensus,$Haplome_1$), and PI(Consensus,$Haplome_2$) for each chromosome in MGA and hifiasm consensus assemblies of the RUST and RUST-Pst datasets. Each PI is provided in the format PI_1/PI_2, where PI_1 is the standard percent identity and PI_2 is the percent identity that does not account for large gaps ≥10 bp. For each chromosome of each dataset, the PI_1/PI_2 with the highest PI_1 is in bold. Pairwise alignments were generated using UniAligner as minimap2 has limitations in generating alignments across entire chromosomes (Supplementary Note 11). When multiple contigs align to the same haplome, the largest contig is retained for the haplome. We use the term "consensus PI" to denote the larger of PI_1(Consensus,$Haplome_1$) and PI_1(Consensus,$Haplome_2$). For RUST, MGA generated higher consensus PIs for chromosomes 6, 7, 10, 12, 13, 14, 16, 17, and 18 while hifiasm generated higher consensus PIs for chromosomes 1, 2, 4, 5, 8, and 11. For RUST-Pst, MGA generated higher consensus PIs for chromosomes 3, 4, 7, 8, 9, 10, 11, and 17 while hifiasm generated higher consensus PIs for chromosomes 5, 6, 14, 16, and 18.

Supplementary Note 10: Exploring short contigs in MGA assemblies

Table S7 summarizes the annotation of short contigs based on BlastN (v2.16.0+) [18] against the NT database (updated 2025/06/21) for RUST, RUST-Pst, and BONOBO dataset. In RUST, four short contigs and one long contig of 1.6 Mb were mapped to *Herbaspirillum hiltneri* N3, a bacterium associated with wheat roots [19]. A contig of 40,761 bp was annotated as *Paradevosia shaoguanensis* strain J5-3, reported from wheat feed soil [20]. Another contig of 33,290 bp was mapped to plasmid pXEA29.3836R of *Xanthomonas euvesicatoria* pv. *alfalfae* strain CFBP3836, a pathogen that causes bacterial leaf spot in alfalfa [21]. In RUST-Pst, a contig of 7,707 bp was mapped to *Glutamicibacter* sp. M10, a soil bacterium that can influence plant growth [22]. In BONOBO, a contig of 170,974 bp was identified as Human gammaherpesvirus 4 (EBV), a virus highly prevalent in humans and associated with several cancers [23]. In addition to these bacterial and viral sequences, MGA also assembled the mitochondrial genomes for all three datasets: RUST, RUST-Pst, and BONOBO.

Table S7. Annotation of short essential contigs (<1 Mb) in MGA assemblies for RUST, RUST-Pst, and BONOBO.

Dataset	Contig length	Reference ID	Reference name	Reference length	Query coverage	Percent identity
**RUST**	961391	CP011409.1	*Herbaspirillum hiltneri* N3	4965474	41.65	80.97
**RUST**	336741	CP011409.1	*Herbaspirillum hiltneri* N3	4965474	50.08	80.68
**RUST**	196992	CP011409.1	*Herbaspirillum hiltneri* N3	4965474	60.07	80.87
**RUST**	138772	CP011409.1	*Herbaspirillum hiltneri* N3	4965474	41.61	81.19
**RUST**	75978	NC_044103.1	*Puccinia triticina* isolate HnZU18-3 mitochondrion	77894	99.61	99.57
**RUST**	40761	CP068983.1	*Paradevosia shaoguanensis* strain J5-3 chromosome	4629545	4.55	81.99
**RUST**	33290	CP072269.1	*Xanthomonas euvesicatoria* pv. *alfalfae* strain CFBP3836 plasmid pXEA29.3836R	28841	62.76	91.57
**RUST**	19403	NC_030916.1	*Tsukamurella* phage TPA4	56212	7.81	75.84
**RUST**	12686	OZ001412.1	*Cenchrus americanus* genome assembly, segment: PgSegmented	9861679	100	98.83
**RUST**	12381	OZ012670.1	*Lordithon lunulatus* genome assembly, chromosome: 5	59719488	17.79	91.76
**RUST**	10900	CP110441.1	*Puccinia triticina* strain Pt15 chromosome 3B	8495914	100	96.37
**RUST**	8831	LR722623.1	*Hordeum vulgare* subsp. *vulgare* genome assembly, chromosome: 0H	85026395	62.34	96.56
**RUST**	8602	CP128824.1	*Candida albicans* strain SC5314 chromosome R	2385618	57.67	88.33
**RUST**	7436	CP139824.1	*Streptomyces rochei* strain D21E05 chromosome	8597388	11.92	85.48

**RUST-Pst**	103611	MN746374.1	*Puccinia striiformis* f. sp. *tritici* strain DK0911 mitochondrion	101813	93.46	96.96
**RUST-Pst**	8616	OX637636.1	*Tausonia pullulans* genome assembly, chromosome: 17	647864	64.32	92.17
**RUST-Pst**	7707	CP104918.1	*Glutamicibacter* sp. M10 chromosome	3529741	26.04	93.56
**RUST-Pst**	7632	OZ057406.1	*Allodiplogaster pararmata* genome assembly, chromosome: 4	44858016	39.88	82.54
**RUST-Pst**	6737	CP068570.1	*Phenylobacterium glaciei* strain 20VBR1 chromosome	4270368	29.17	70.81
**BONOBO**	849074	OZ036148.1	*Homo sapiens* genome assembly, chromosome: contig-1	58903441	95.55	92.31
**BONOBO**	707639	CP034500.1	Eukaryotic synthetic construct chromosome 21	38708702	69.39	86.19
**BONOBO**	522305	AC239641.3	*Homo sapiens* BAC clone RP11-1271P18 from chromosome x	166469	67.01	96.73
**BONOBO**	493385	AC270185.1	*Homo sapiens* BAC clone CH17-402N4 from chromosome 11	229008	60.03	92
**BONOBO**	465562	CP139550.2	*Homo sapiens* isolate NA24385 chromosome 16	93613879	71.19	93.77
**BONOBO**	463077	CP139550.2	*Homo sapiens* isolate NA24385 chromosome 16	93613879	43.28	89.78
**BONOBO**	331905	CP068263.2	*Homo sapiens* isolate CHM13 chromosome 15	99753195	78.38	88.71
**BONOBO**	327252	CP139534.2	*Homo sapiens* isolate NA24385 chromosome Y	62432599	98.13	89.58
**BONOBO**	264425	OZ036274.1	*Homo sapiens* genome assembly, chromosome: contig-1	23939036	88.71	91.94
**BONOBO**	216884	AC244099.2	*Homo sapiens* BAC clone CH17-227A24 from chromosome x	202755	68.53	96.08
**BONOBO**	170974	NC_007605.1	Human gammaherpesvirus 4	171823	100	99.88
**BONOBO**	94967	OZ036274.1	*Homo sapiens* genome assembly, chromosome: contig-1	23939036	77.88	92.53
**BONOBO**	28415	AC186878.3	*Pan troglodytes* BAC clone CH251-633J4 from chromosome 4, complete sequence	207766	100	91.74
**BONOBO**	22836	AP023495.1	*Homo sapiens* DNA, sequence_id: unplaced_0010	474654	100	76.68
**BONOBO**	22336	CP139521.2	*Homo sapiens* isolate NA24385 chromosome 21	47311724	100	75.11
**BONOBO**	16569	HM015213.1	*Pan paniscus* isolate PP25 mitochondrion	16569	100	100
**BONOBO**	8521	AC275315.1	*Pan troglodytes* chromosome 8 clone CH251-16F3	168793	100	91.32
**BONOBO**	6438	OX104059.1	*Dicrocoelium dendriticum* genome assembly, chromosome: 10	81857327	99.74	80.39
**BONOBO**	6313	OX104070.1	*Dicrocoelium dendriticum* genome assembly, chromosome: 8	89101726	99.71	89.59
**BONOBO**	6299	OV121137.1	*Brassicogethes aeneus* genome assembly, chromosome: 6	56578884	100	87.12
**BONOBO**	6068	OX103911.1	*Schistosoma rodhaini* genome assembly, chromosome: 1	86950095	99.95	83.62

The annotations were generated by running BlastN against the NT database. For contigs aligned to multiple reference sequences, only the one with the largest query coverage is shown. Short contigs not aligned to any reference were excluded from this Table.

Supplementary Note 11: Dot plots

- Fig. S14 presents UniAligner dot plots of the consensus contig of RUST chromosome 3 against the corresponding haplomes 1 and 2 in $Genome_{RUST}$.
- Fig. S15-S20 present UniAligner dot plots of the MGA consensus contigs of RUST, RUST-Pst, BONOBO, HUMAN, GIRAFFE, and SHEEP datasets against the corresponding haplomes (suffixes A/B denote haplomes 1/2; suffixes M/P denote paternal/maternal haplomes).

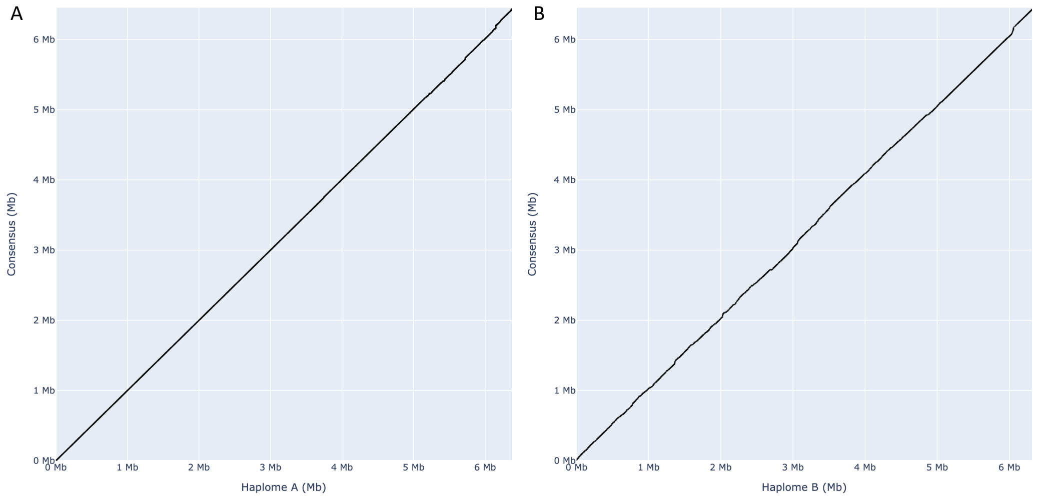

Fig. S14. UniAligner dot plots compare a single MGA consensus contig (chromosome 3 in the *Pt* genome) in the RUST dataset assembly against the two *Pt* haplomes 1 and 2 of chromosome 3 in Genome$_{\mathbf{RUST}}$. The dot plots illustrate that the suffix of the consensus contig of RUST chromosome 3 aligns well with the suffix of haplome 1/2 (i.e., haplome A/B in this Figure) of chromosome 3. In contrast, using minimap2 (version 2.21-r1071) with the options "asm20" and "-p 0.1" to align consensus chromosome 3 (query) to haplome 1 (reference) independently produced an alignment spanning 0–95% of the query to 0.00–95.98% of the reference, with percent identity of 99/100%. Aligning consensus chromosome 3 (query) to haplome 2 (reference) independently yielded an alignment spanning 77–94% of the query to 77.44–95.41% of the reference, with percent identity of 97/100%. However, aligning only the 100 kb suffix of consensus chromosome 3 (query) to the 100 kb suffix of haplome 2 (reference) using minimap2 produced a full-length alignment spanning 0–100% of the query to 0.03–100% of the reference, with percent identity of 100/100%, suggesting that minimap2 may be less effective for chromosome-length sequence alignments.

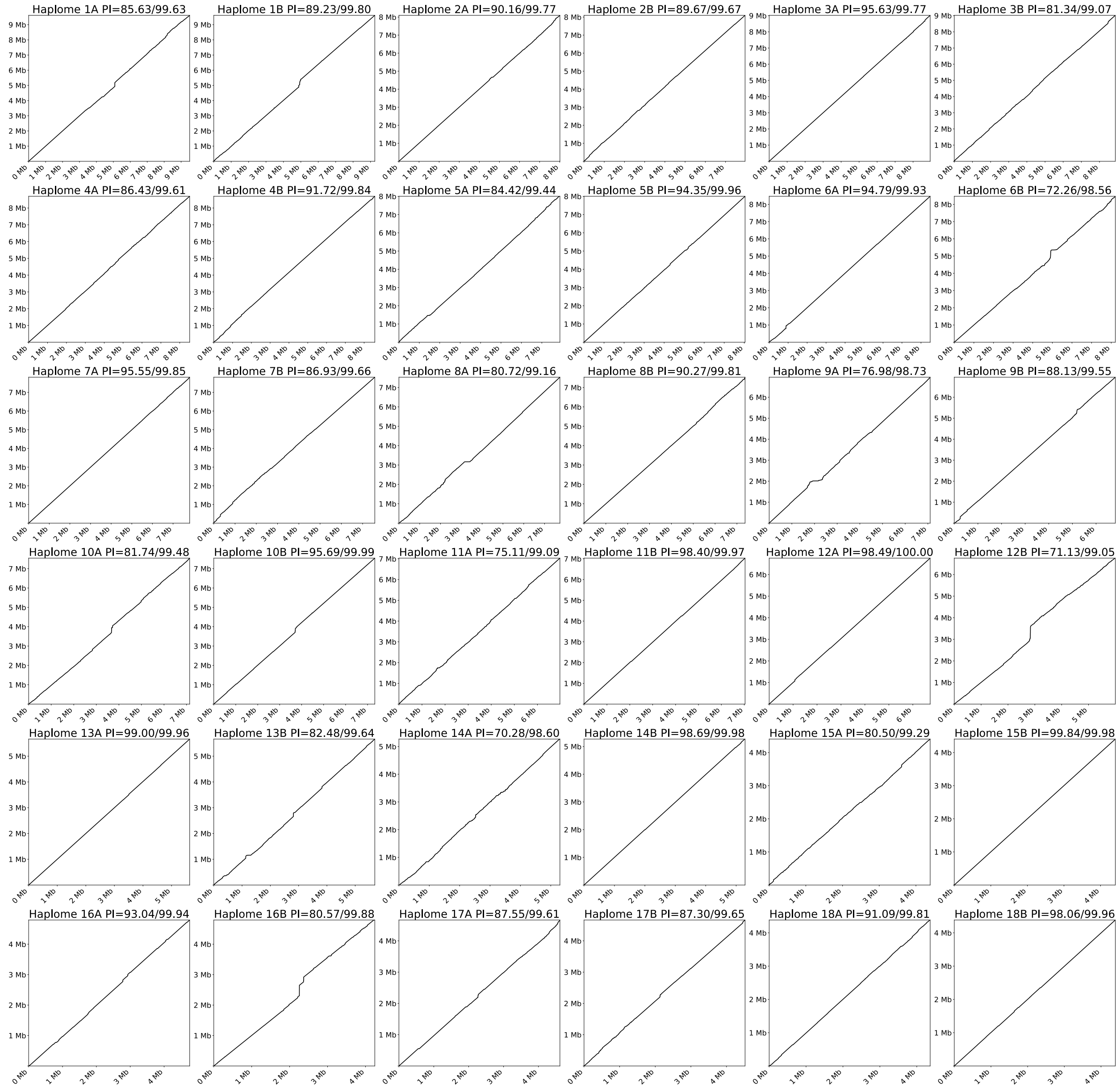

Fig. S15. UniAligner dot plots compare MGA consensus contigs from the RUST dataset against the *Pt* haplomes. Each subplot displays the alignment between a consensus contig (y-axis) and a specific chromosome haplome (x-axis). Alignment quality is indicated by PI_1/PI_2, representing standard percent identity and identity ignoring large gaps ≥10 bp, respectively. The plots demonstrate high similarity, with each consensus contig aligning closely to at least one of the two haplomes. Note that some inconsistencies might originate from the gaps in the diploid assembly $Genome_{RUST}$, where the 18×2 haplomes are connected from 289 gapless contigs.

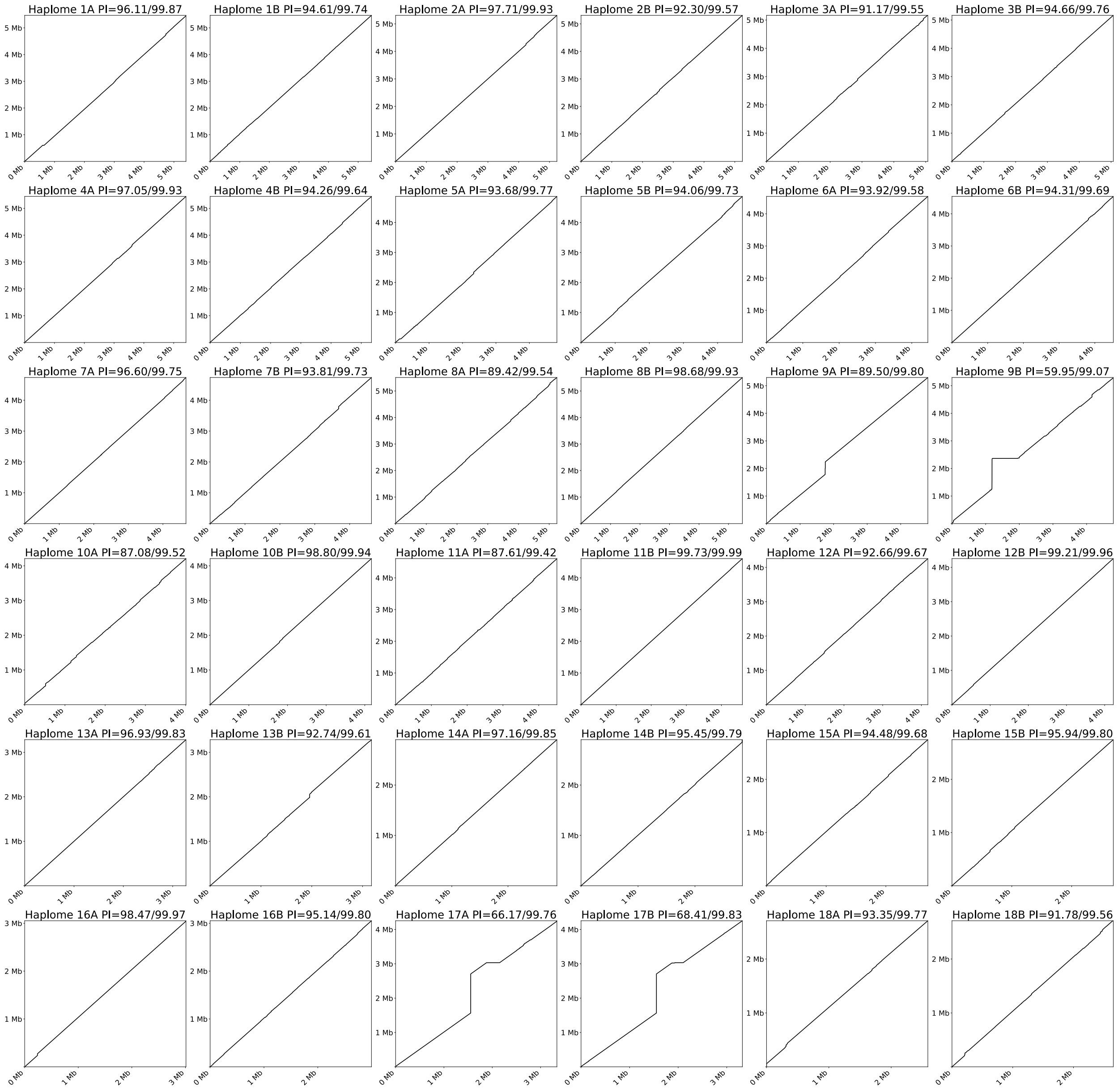

Fig. S16. UniAligner dot plots compare MGA consensus contigs from the RUST-Pst dataset against the *Pst* haplomes. Each subplot displays the alignment between a consensus contig (y-axis) and a specific chromosome haplome (x-axis). Alignment quality is indicated by PI_1/PI_2, representing standard percent identity and identity ignoring gaps ≥10 bp, respectively. The plots demonstrate high similarity, with each consensus contig aligning closely to at least one of the two haplomes, except for chromosomes 9 and 17. Note that haplomes 9A, 17A, and 17B in $\mathrm{Genome}_{\mathrm{RUST\text{-}Pst}}$ have gaps at positions 1.8 Mb, 1.9 Mb, and 1.9 Mb, respectively.

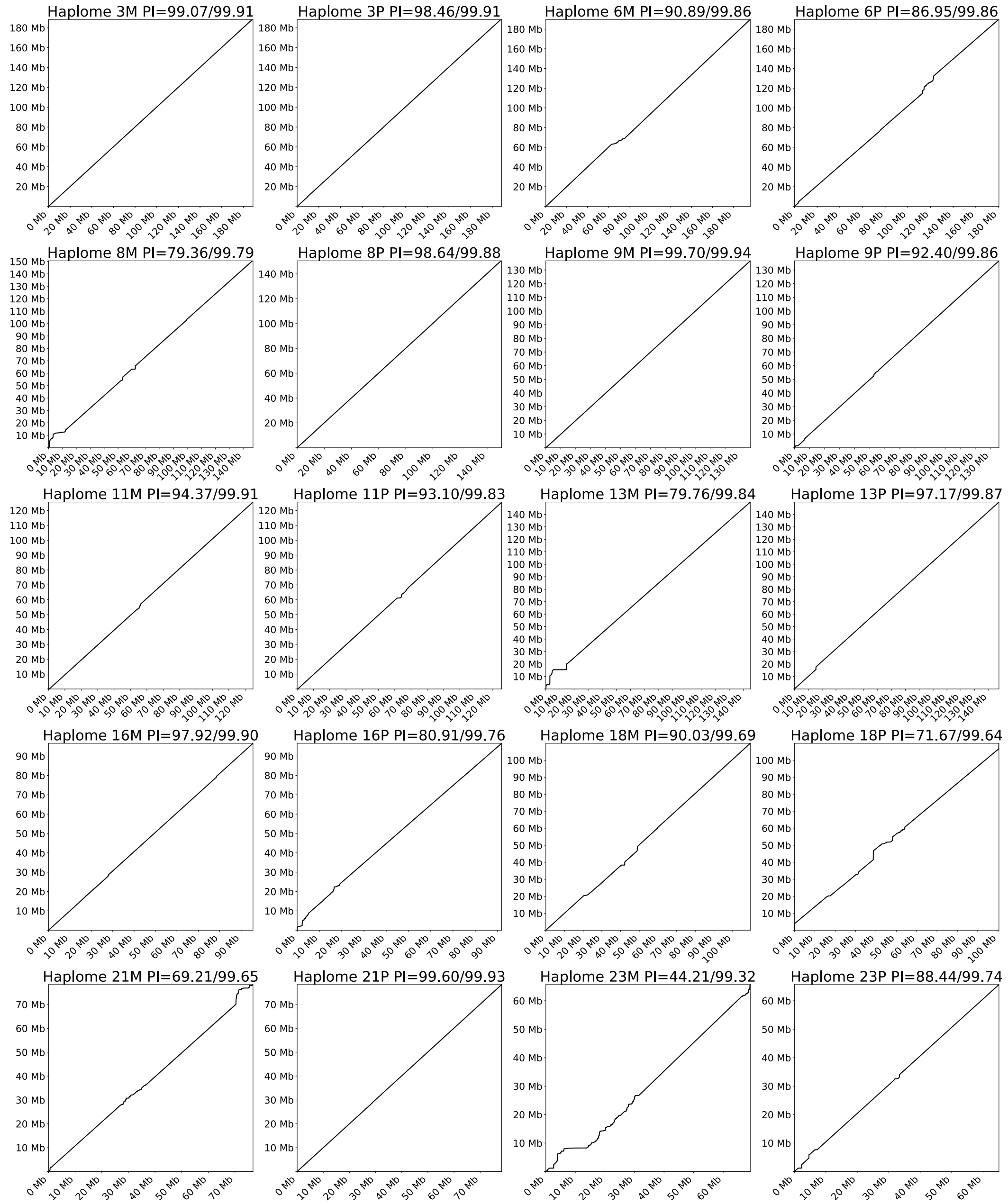

Fig. S17. UniAligner dot plots compare MGA consensus contigs from the BONOBO dataset against their respective bonobo haplomes. Each subplot displays the alignment between a consensus contig (y-axis) and a specific chromosome haplome (x-axis). Alignment quality is indicated by PI_1/PI_2, representing standard percent identity and identity ignoring gaps ≥10 bp, respectively. Note that the T2T haplome 23M has an unassembled gap from 1,616,720 to 2,616,721, while the T2T haplome 23P has an unassembled gap from 1,258,553 to 2,258,554 (annotated as "Centromeric Satellite Annotation" from the UCSC Genome Browser on Pan paniscus mPanPan1 v2.0 (Bonobo) (mPanPan1_v2.0)).

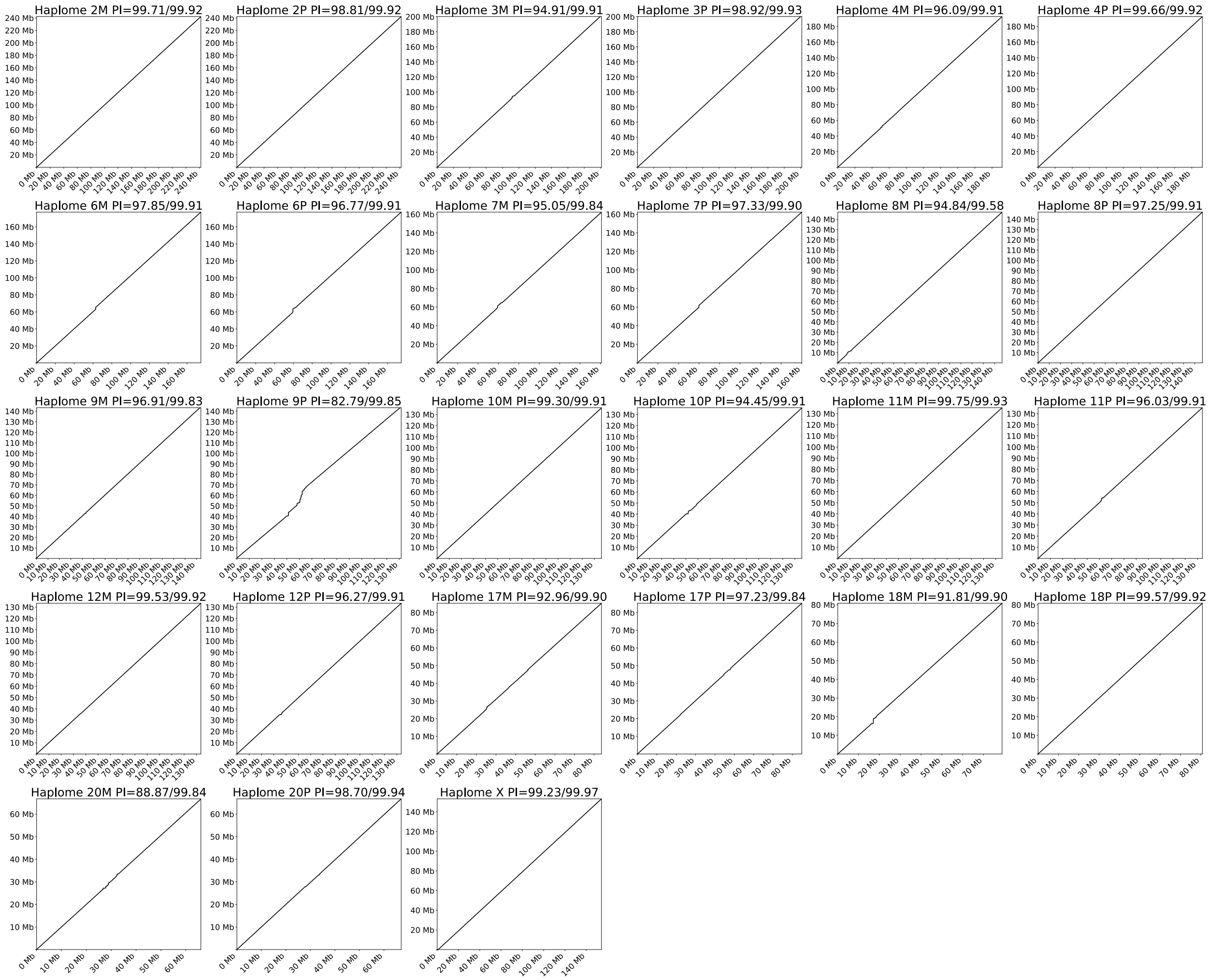

Fig. S18. UniAligner dot plots compare MGA consensus contigs from the HUMAN dataset against their respective human haplomes. Each subplot displays the alignment between a consensus contig (y-axis) and a specific chromosome haplome (x-axis). Alignment quality is indicated by PI_1/PI_2, representing standard percent identity and identity ignoring gaps ≥10 bp, respectively. The plots demonstrate high similarity, with each consensus contig aligning closely to at least one of the two haplomes.

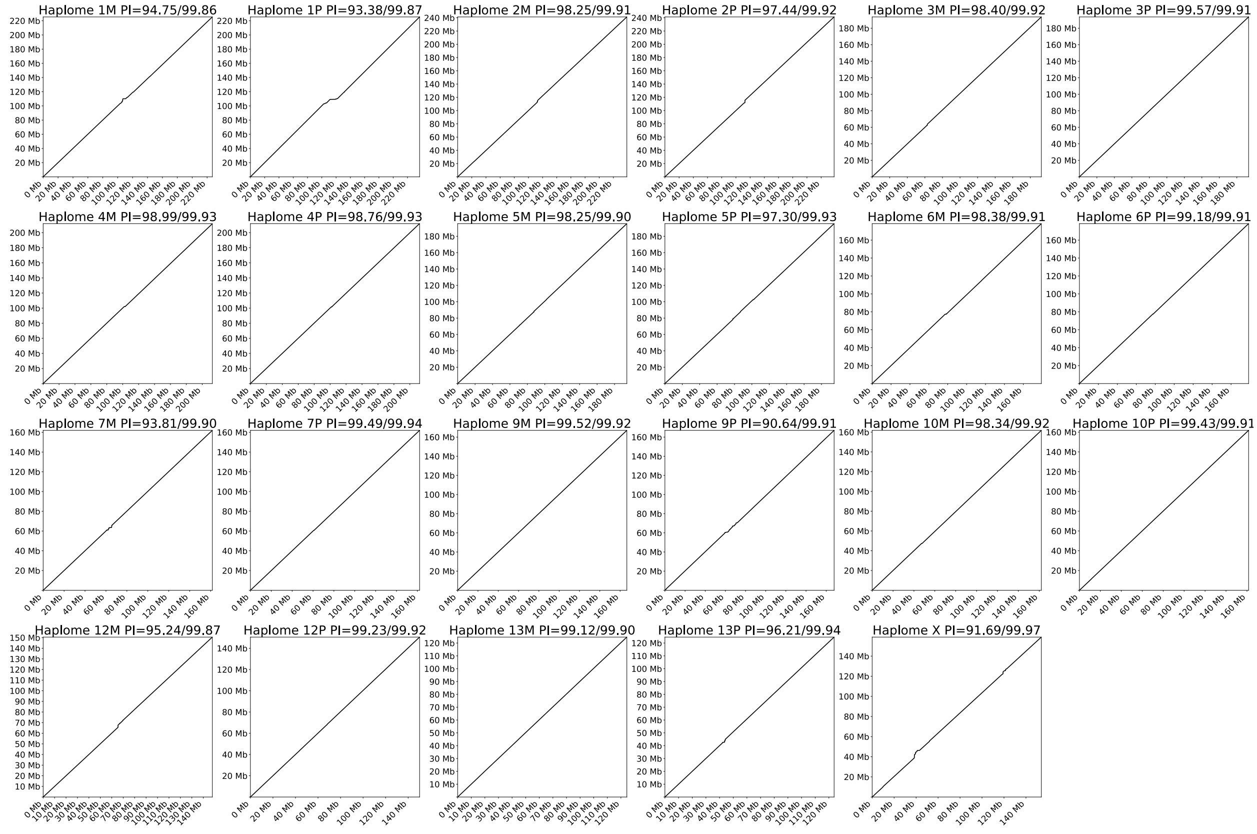

Fig. S19. UniAligner dot plots compare MGA consensus contigs from the GIRAFFE dataset against their respective giraffe haplomes, with suffixes M/P denoting paternal/maternal origin. Each subplot displays the alignment between a consensus contig (y-axis) and a specific chromosome haplome (x-axis). Alignment quality is indicated by PI_1/PI_2, representing standard percent identity and identity ignoring gaps ≥10 bp, respectively. The plots demonstrate high similarity, with each consensus contig aligning closely to at least one of the two haplomes.

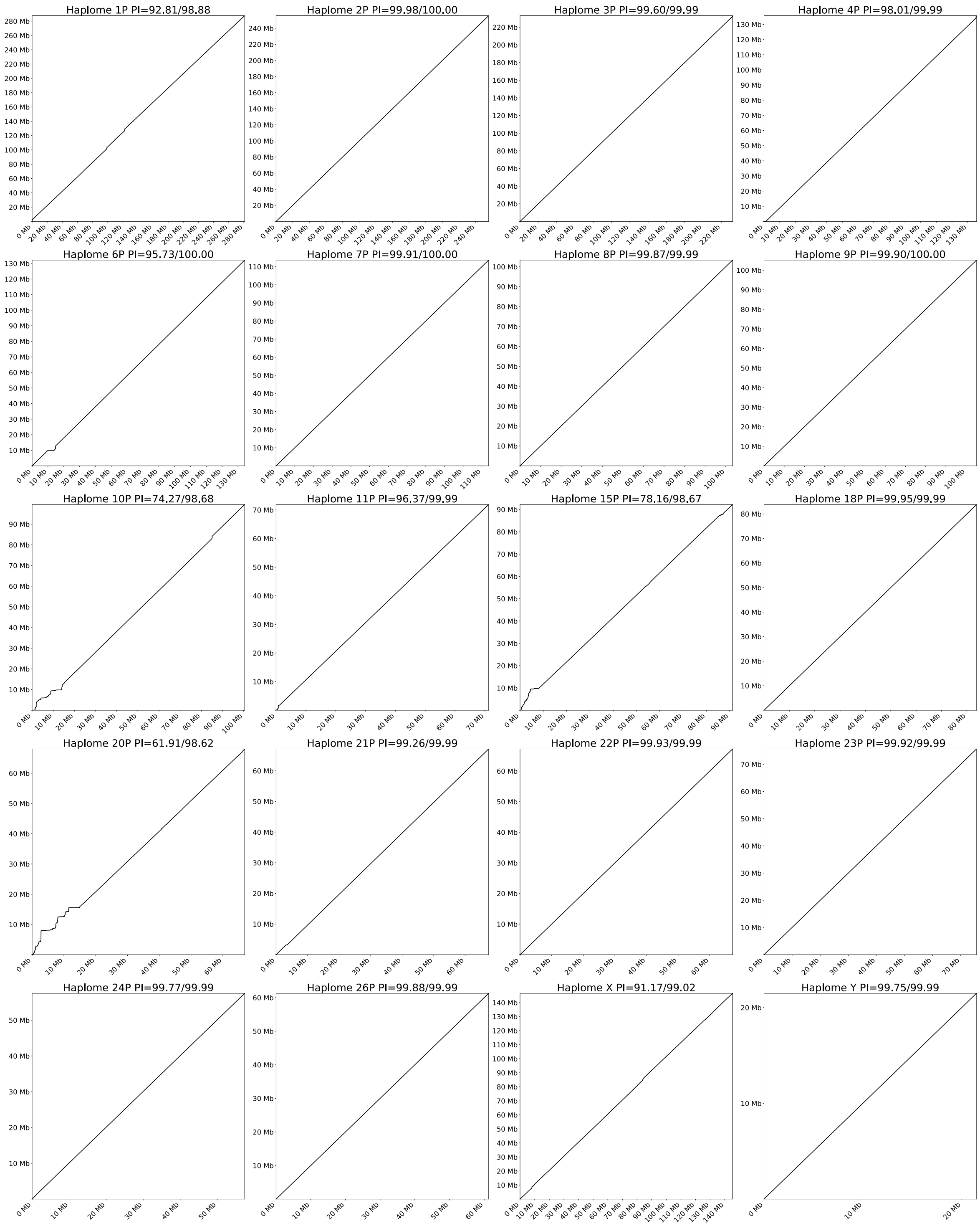

Fig. S20. UniAligner dot plots compare MGA consensus contigs from the SHEEP dataset against their respective sheep paternal haplomes. Each subplot displays the alignment between a consensus contig (y-axis) and a specific chromosome haplome (x-axis). Alignment quality is indicated by PI_1/PI_2. For many chromosomes, the plots demonstrate high similarity of the consensus contig aligning closely to the paternal haplome. Some inconsistencies can reflect the fact that the consensus contig incorporates some segments of the still unassembled maternal haplome.

Supplementary Note 12: Hifiasm assembly of bonobo chromosome 22

Hifiasm assembled chromosome 22 into a single homopolymer-uncompressed long contig of length 46.7 Mb, with 1-100% of the contig aligning to chromosome 22P spanning 0.37%-99.99%. Below, we raise concerns about the validity of the hifiasm assembly of chromosome 22 into a single contig, because chromosomes 22 and 14 share a long perfect repeat that is not bridged by any reads.

MGA assembled chromosome 22 into two long homopolymer-uncompressed contigs: one of length 48.6 Mb, with 7%-100% of the contig aligning to chromosome 22P spanning 5.51%-99.99%, and another of 1.7 Mb, with 31%-95% of the contig aligning to chromosome 22P spanning 0.37%-2.55%. The start of the longer MGA contig contains complex difficult-to-align repeat sequences. Figure 1 (homopolymer-compressed) suggests that chromosome 22P shares a long, nearly perfect repeat with chromosomes 14, 15, 17, and 23 (compressed/uncompressed length 48,357/68,803 bp). We analyzed this repeat (R) in detail on the homopolymer-uncompressed chromosomes 22P and 14P (denoted R_{22} and R_{14}).

The repeat R spans positions R_{22}=2,380,972-2,449,776 bp (4.91%-5.05%) on chromosome 22 with PI_1/PI_2=100.00/100.00 and spans positions R_{14}=2,830,416-2,899,221 bp (2.44%-2.50%) on chromosome 14 with PI_1/PI_2=99.99/99.99. The UniAligner global alignment between R_{22} and R_{14} yielded PI_1/PI_2=99.99/99.99 and the following CIGAR string:

15962M **1X** 14005M **1X** 5185M **1D** 1077M **1X** 9027M **1X** 23544M

This alignment reveals a perfect repeat of 23,544 bp shared between 22P and 14P—spanned by no HiFi reads in the BONOBO dataset. Consequently, it is theoretically impossible to assemble chromosome 22P into a single contig without making risky repeat-resolution decisions, which are prone to introducing assembly errors.

Supplementary Note 13: Consensus Genome algorithm

Below we provide the pseudocode for the Genome Consensus (CG) algorithm:

CG(*Graph*)
  while there are valid detours or valid whirls in *Graph*
    while there are valid detours in *Graph*
      Detour ← a detour with the highest PI among all shortest valid detours in *Graph*
      Graph ← **Detouring**(*Graph*,*Detour*)
    while there are valid whirls in *Graph*
      Whirl ← a shortest valid whirl in *Graph*
      Graph ← **Dewhirling**(*Graph*,*Whirl*)
  return *Graph*

Supplementary Note 14: Inferring read coverage of edges in the multiplex de Bruijn graph

The MDB graph *multiplexDB*=multiDB$_k$(*Reads**) is subjected to various simplification procedures that utilize the read coverage. Since LJA does not compute the read coverages of

edges in the MDB graph, we implemented a module that computes these coverages in MGA. Each edge *e*=(*v,w*) in the graph *multiplexDB* corresponds to a path *Path*(*e*) in the graph *DB*=DB_k(*Reads**). The function **InferringCoverage**(*multiplexDB,DB,e*) constructs the path *Path*(*e*) by aligning the sequence of *e* to the graph *DB* and inferring the read coverage of *e* in *multiplexDB* as the minimum read coverage of all edges longer than or equal to 100 kb in *Path*(*e*). If all edges in *Path*(*e*) are shorter than 100 kb, the coverage of *e* is defined as the minimum coverage of all edges in this path. The function **InferringCoverage**(*multiplexDB,DB*) applies **InferringCoverage**(*multiplexDB,DB,e*) to all edges of the graph *multiplexDB*. The edge multiplicities in the MDB graph are inferred using the same approach as in LJA_k(*Reads*).

Supplementary Note 15: Repairing broken paths

Given a *distance parameter d*, a paths $v,v_1,...,v_{t-1},v_t$ is called *d-constrained* if$|seq(v,v_1,...,v_{t-1},v_t)| > d$ but $|seq(v, v_1,...,v_{t-1})| \leq d$. Given a node *v* in a graph, we define $Paths_v(d)$ as the set of all *d*-constrained paths starting at *v*. For each out-tip (*v,w*), MGA constructs the path-set $Paths_v(|seq(v,w)|)$ and identifies a path *RepairPath*(*v,w*) in this path-set that maximizes the score of the prefix alignment between seq(*RepairPath*(*v,w*)) and seq(*v,w*). If the percent identity of this alignment exceeds a threshold *minRepairPI* (default 80%), we classify *RepairPath*(*v,w*) as *valid* and denote its last edge as *RepairEdge*(*v,w*). Similarly, for each in-tip (*u,t*), we define *RepairPath*(*u,t*) and *RepairEdge*(*u,t*) using the suffix alignment.

MGA classifies an out-tip (*v,w*) and an in-tip (*u,t*) as a *tip-pair* if their repair-edges are identical and defines *RepairPath*(*v,t*) as the combined path of *RepairPath*(*v,w*) and *RepairPath*(*u,t*). Given a tip-pair formed by an out-tip (*v,w*) and an in-tip (*u,t*) in an MDB graph *G*, the *repairing operation* repairs the broken edge (*v,t*) that generated this tip-pair by removing tips (*v,w*) and (*u,t*) and increasing the coverage of edges in *RepairPath*(*v,t*) by the average coverage of the tips (*v,w*) and (*u,t*). MGA further extends the repairing operation to all out-tips and in-tips that are not in tip-pairs. Such tips often represent a special case of a broken edge, e.g., when an out-tip (*v,w*) is present in the graph but an in-tip (*u,t*) is not. Given the out-tip (*v,w*) in an MDB graph *G*, MGA identifies *RepairPath*(*v,w*) as described above, increases the coverage of all its edges by cov(*v,w*), and removes the out-tip (*v,w*) (in-tips are repaired similarly).

When MGA finds an out-tip (*v,w*), it first tries to repair it using the function **RepairingTips**. But this operation may fail because a *valid RepairPath*(*v,w*) may not exist. This might happen because (*v,w*) and *RepairPath*(*v,w*) belong to two different chromosomes, or to two highly-heterozygous regions within the same chromosome. To repair such tips, we extend the **RepairingTips** function by the **RepairingTips+** function that is launched after the **Connect** operation and before the **Deduplication** operation.

RepairingTips+ simplifies the *Y-subgraph* formed by three edges incident to a node *w* with outdegree(*w*)=1 and indegree(*w*)=2 (Fig. S21). Two of these edges enter into a node *w* and one exits this node: an edge $in_1=(v_1,w)$; an edge $in_2=(v_2,w)$ where $v_1 \neq v_2$; and an edge *out*=(*w,o*) where outdegree(*o*)=0, indegree(*o*)=1. We assume that the length of edge in_2 does not exceed the length

of the edge in_1. Edges in_1 and in_2 are either traversed by two haplotypes of the same chromosome (and thus represent diverged haplotypes) or traversed by two different chromosomes.

Figure S21 illustrates three operations on the Y-subgraph $Y=(in_1, in_2, out)$:

- Remove(Y) (Fig. S21A) removes the edge in_2 from the graph.
- Split(Y) (Fig. S21B) adds two new nodes (o' and o''), and replaces three edges in_1, in_2 and out by two edges (v_1,o') and (v_2,o''), where (v_1,o') spells the sequence of the path (v_1,w,o) and (v_2,o'') spells the sequence of the path (v_2,w,o).
- Detach(Y) (Fig. S21C) adds a new node o', removes edges in_1 and out, and adds an edge (v_1,o') that spells the sequence of the path (v_1,w,o).

Below we analyze the Y-subgraph $Y=(in_1, in_2, out)$ and consider three cases:

- Case 1 (Fig. S22A): both edges $in_1=(v_1,w)$ and $in_2=(v_2,w)$ are in-tips, i.e., outdegree(v_1)=outdegree(v_2)=1 and indegree(v_1)=indegree(v_2)=0: If the 1 Mb suffixes of edges in_1 and in_2 are similar (a percent identity of at least PI_{Tip} with default 60%), we assume that they are traversed by two haplotypes of the same chromosome and perform operation Remove(Y). Otherwise, we assume that they are traversed by different chromosomes and consider two situations depending on whether the edge $out=(w,o)$ is long (has length at least *SharedLength*) or short. If it is long, no action is taken. Otherwise, we classify this edge as traversed by both chromosomes and perform operation Split(Y).
- Case 2 (Fig. S22B): the edge $in_1=(v_1,w)$ is an in-tip (outdegree(v_1)=1 and indegree(v_1)=0) but the edge $in_2=(v_2,w)$ is not an in-tip (indegree(v_2)>0).
    1. If there exists a valid *RepairPath*(v_1,w), repair the tip (v_1,w) using the operation **RepairingTips**.
    2. Otherwise, we assume that edges in_1 and in_2 are traversed by different chromosomes Chr_1 and Chr_2 and consider two possible situations depending on whether the edge $out=(w,o)$ is long or short. If it is long, we assume that it belongs to only one chromosome, (because node v_1 is a tip but node v_2 is not), and perform operation Detach (Y). Otherwise, we classify it as a shared edge between two chromosomes and perform operation Split(Y).
- Case 3 (Fig. S22C): both edges $in_1=(v_1,w)$ and $in_2=(v_2,w)$ are non-tips:
    1. If the edge $out=(w,o)$ is sufficiently short (shorter than *SufficientShort*, default 200 kb), we assume that it is traversed by both chromosomes (whether they are the same or not), and perform Split(Y).
    2. Otherwise, no action is taken (the graph remains complex).

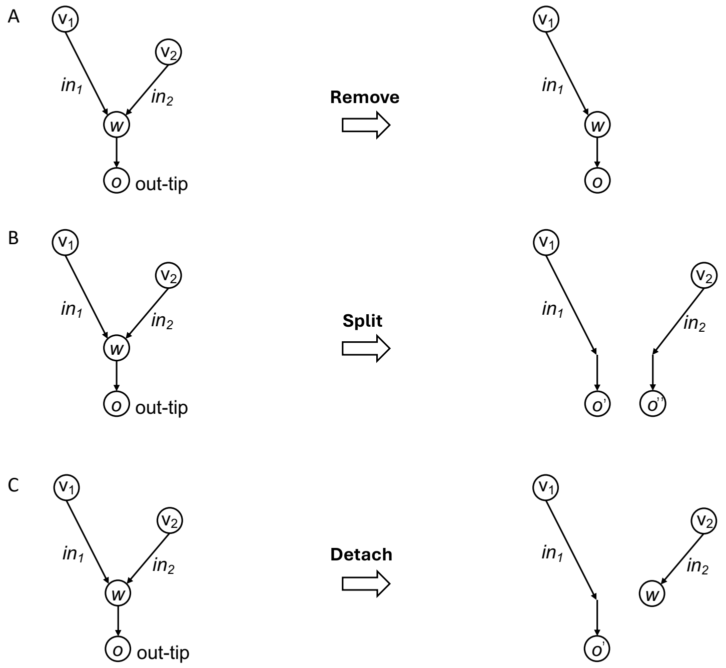

Fig. S21. Remove, Split, and Detach operations on the Y-subgraph. Even though nodes v_1 and v_2 are shown as out-tips, they are not necessarily out-tips (Fig. S23).

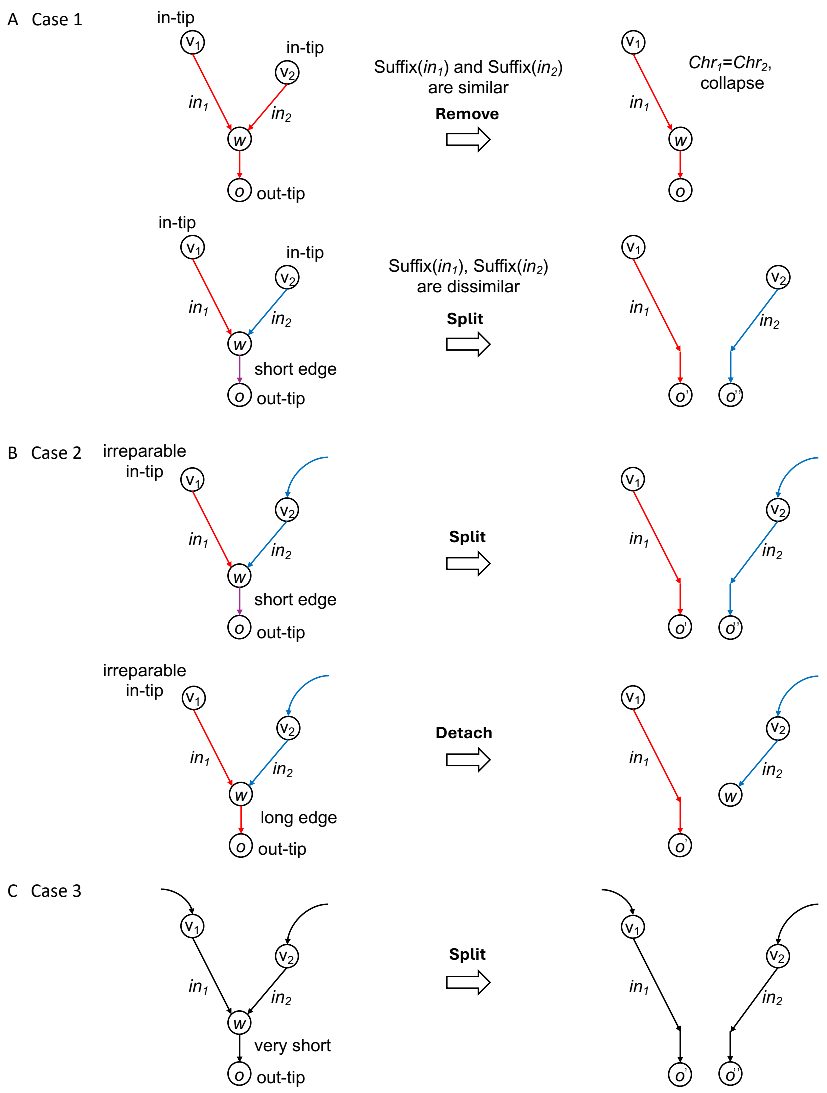

Fig. S22. Analysis of various cases for the function RepairingTips+. Red and blue edges belong to different "red" and "blue" chromosomes. Purple edges represent shared edges of red and blue chromosomes. Black edges represent edges for which chromosome assignments are unknown and are not important.

Supplementary Note 16: Estimating heterozygosity rate

We estimate the heterozygosity rate of a diploid genome by aligning its two haplomes using minimap2 (version 2.21-r1071) with "asm5" and "--cs" options and generating a PAF alignment file. Variants are called from the PAF file using the paftools.js tool (provided with minimap2) with default parameters. We retain only variants that are covered exactly once by the query sequence and have high mapping quality (MAPQ=60). The heterozygosity rate is calculated as the number of retained variants divided by the total number of bases across all callable regions in the output file of paftools.js.

References

1. Antipov D, Rautiainen M, Nurk S, Walenz BP, Solar SJ, Phillippy AM, et al. Verkko2 integrates proximity-ligation data with long-read De Bruijn graphs for efficient telomere-to-telomere genome assembly, phasing, and scaffolding. Genome Research. 2025;35:1583–94. https://doi.org/10.1101/gr.280383.124

2. Pevzner PA, Tang H, Tesler G. De novo repeat classification and fragment assembly. Genome Res. 2004;14:1786–96. https://doi.org/10.1101/gr.2395204

3. Yoo D, Rhie A, Hebbar P, Antonacci F, Logsdon GA, Solar SJ, et al. Complete sequencing of ape genomes. Nature. 2025;641:401–18. https://doi.org/10.1038/s41586-025-08816-3

4. Kim J, Rosen BD, Fumagalli SE, Kuhn KL, Long A, Schoenebeck JJ, et al. Finishing a complete giraffe genome from telomere to telomere with Verkko-Fillet. bioRxiv. 2025;2025.10.01.679366. https://doi.org/10.1101/2025.10.01.679366

5. Olagunju TA, Pospelova M, Schwartz JC, Mousel MR, Piel LMW, Grossman PC, et al. Bighorn sheep T2T genome assembly reveals differences in immune genes: a potential cause of high morbidity due to respiratory pathogens. bioRxiv. 2025;2025.09.30.679298. https://doi.org/10.1101/2025.09.30.679298

6. Seah BKB, Singh A, Vetter DE, Emmerich C, Peters M, Soltys V, et al. Nuclear dualism without extensive DNA elimination in the ciliate *Loxodes magnu*s. Proceedings of the National Academy of Sciences. 2024;121:e2400503121. https://doi.org/10.1073/pnas.2400503121

7. Cheng H, Concepcion GT, Feng X, Zhang H, Li H. Haplotype-resolved de novo assembly using phased assembly graphs with hifiasm. Nat Methods. 2021;18:170–5. https://doi.org/10.1038/s41592-020-01056-5

8. Koren S, Rhie A, Walenz BP, Dilthey AT, Bickhart DM, Kingan SB, et al. De novo assembly of haplotype-resolved genomes with trio binning. Nat Biotechnol. 2018;36:1174–82. https://doi.org/10.1038/nbt.4277

9. Rautiainen M, Marschall T. GraphAligner: rapid and versatile sequence-to-graph alignment. Genome Biol. 2020;21:253. https://doi.org/10.1186/s13059-020-02157-2

10. Duan H, Jones AW, Hewitt T, Mackenzie A, Hu Y, Sharp A, et al. Physical separation of haplotypes in dikaryons allows benchmarking of phasing accuracy in Nanopore and HiFi assemblies with Hi-C data. Genome Biol. 2022;23:84. https://doi.org/10.1186/s13059-022-02658-2

11. Mikheenko A, Prjibelski A, Saveliev V, Antipov D, Gurevich A. Versatile genome assembly evaluation with QUAST-LG. Bioinformatics. 2018;34:i142–50. https://doi.org/10.1093/bioinformatics/bty266

12. Ye C, Ma ZS, Cannon CH, Pop M, Yu DW. Exploiting sparseness in de novo genome assembly. BMC Bioinformatics. 2012;13:S1. https://doi.org/10.1186/1471-2105-13-S6-S1

13. Li K, Xu P, Wang J, Yi X, Jiao Y. Identification of errors in draft genome assemblies at single-nucleotide resolution for quality assessment and improvement. Nat Commun. 2023;14:6556. https://doi.org/10.1038/s41467-023-42336-w

14. Gurevich A, Saveliev V, Vyahhi N, Tesler G. QUAST: quality assessment tool for genome assemblies. Bioinformatics. 2013;29:1072–5. https://doi.org/10.1093/bioinformatics/btt086

15. Simão FA, Waterhouse RM, Ioannidis P, Kriventseva EV, Zdobnov EM. BUSCO: assessing genome assembly and annotation completeness with single-copy orthologs. Bioinformatics. 2015;31:3210–2. https://doi.org/10.1093/bioinformatics/btv351

16. Li H. Minimap2: pairwise alignment for nucleotide sequences. Bioinformatics. 2018;34:3094–100. https://doi.org/10.1093/bioinformatics/bty191

17. Bzikadze AV, Pevzner PA. UniAligner: a parameter-free framework for fast sequence alignment. Nat Methods. 2023;20:1346–54. https://doi.org/10.1038/s41592-023-01970-4

18. Chen Y, Ye W, Zhang Y, Xu Y. High speed BLASTN: an accelerated MegaBLAST search tool. Nucleic Acids Res. 2015;43:7762–8. https://doi.org/10.1093/nar/gkv784

19. Rothballer M, Schmid M, Klein I, Gattinger A, Grundmann S, Hartmann A. *Herbaspirillum hiltneri* sp. nov., isolated from surface-sterilized wheat roots. Int J Syst Evol Microbiol. 2006;56:1341–8. https://doi.org/10.1099/ijs.0.64031-0

20. Wang Y, Zhang HH, Zhao C, Han YT, Liu YC, Zhang XL. Isolation and characterization of a novel deoxynivalenol-transforming strain *Paradevosia shaoguanensis* DDB001 from wheat field soil. Lett Appl Microbiol. 2017;65:414–22. https://doi.org/10.1111/lam.12790

21. Yaripour Z, Mohsen Taghavi S, Osdaghi E, Lamichhane JR. Host range and phylogenetic analysis of *Xanthomonas alfalfae* causing bacterial leaf spot of alfalfa in Iran. Eur J Plant Pathol. 2018;150:267–74. https://doi.org/10.1007/s10658-017-1271-0

22. Hidri R, Metoui-Ben Mahmoud O, Zorrig W, Azcon R, Abdelly C, Debez A. The halotolerant rizhobacterium *Glutamicibacter* sp. *alleviates* salt impact on Phragmites australis by producing exopolysaccharides and limiting plant sodium uptake. Plant Direct. 2023;7:e535. https://doi.org/10.1002/pld3.535

23. Santpere G, Darre F, Blanco S, Alcami A, Villoslada P, Mar Albà M, et al. Genome-Wide Analysis of Wild-Type Epstein–Barr Virus Genomes Derived from Healthy Individuals of the 1000 Genomes Project. Genome Biol Evol. 2014;6:846–60. https://doi.org/10.1093/gbe/evu054
